# Direct Quantification of Protein–Protein Interactions in Living Bacterial Cells

**DOI:** 10.1002/advs.202414777

**Published:** 2025-03-24

**Authors:** Soojung Yi, Eunji Kim, Sora Yang, Gyeongmin Kim, Da‐Woon Bae, Se‐Young Son, Bo‐Gyeong Jeong, Jeong Seok Ji, Hyung Ho Lee, Ji‐Sook Hahn, Sun‐Shin Cha, Yeo Joon Yoon, Nam Ki Lee

**Affiliations:** ^1^ Department of Chemistry Seoul National University 1 Gwanak‐ro, Gwanak‐gu Seoul 08826 Republic of Korea; ^2^ Natural Products Research Institute College of Pharmacy Seoul National University 1 Gwanak‐ro, Gwanak‐gu Seoul 08826 Republic of Korea; ^3^ Department of Chemical and Biological Engineering Institute of Chemical Processes Seoul National University 1 Gwanak‐ro, Gwanak‐gu Seoul 08826 Republic of Korea; ^4^ Department of Chemistry and Nanoscience Ewha Womans University 52 Ewhayeodae‐gil, Seodaemun‐gu Seoul 03760 Republic of Korea

**Keywords:** biosensor, FRET, living cells, protein–protein interactions, synthetic biology

## Abstract

Quantitative measurement of protein–protein interactions (PPIs) within living cells is vital for understanding their cellular functions at the molecular level and for applications in synthetic biology, protein engineering, and drug discovery. Although several techniques have been developed to measure PPI strength in vitro, direct measurement of PPI strength within living bacterial cells remains challenging. Here, a method for quantitatively measuring PPIs by determining the dissociation constant (*K*_d_) in living *E. coli* using fluorescence resonance energy transfer (FRET), a technique termed KD‐FRET, is reported. It is found that the direct excitation of the acceptor fluorophore among spectral crosstalks primarily results in non‐interacting pairs exhibiting an apparent *K*_d_, leading to false‐positive signals. KD‐FRET proves highly effective in quantifying various PPI *K*_d_ values, including both heterologous and homologous pairs. Moreover, KD‐FRET enables the quantification of *K*_d_ for interaction pairs that are unmeasurable in vitro owing to their instability under standard buffer conditions. KD‐FRET is successfully applied in the development of a novel synthetic biology tool to enhance naringenin production in *E. coli* and lycopene production in *S. cerevisiae* by precisely engineering metabolic pathway. These results demonstrate the potential of KD‐FRET as a powerful tool for studying PPIs in their native cellular environments.

## Graphical abstract

Quantitative measurement of protein–protein interactions in living cells is vital for biotechnology applications. Fluorescence resonance energy transfer (FRET) signals in bacterial cells are often weak, leading to false‐positive. Quantitative FRET measurements are implemented to overcome the weak FRET signal. The dissociation constants of various protein–protein pairs, which are subsequently applied to engineer metabolic pathways, are accurately quantified.

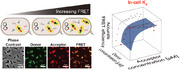

## 1 Introduction

The exploration of protein–protein interactions (PPIs) is a cornerstone of cellular biology, guiding essential processes that underpin various cellular functions.^[^ ^1^ ^]^ Understanding the strength of binding interactions between proteins is crucial for elucidating cellular mechanisms, disease pathways, and developing therapeutic interventions targeting PPIs.^[^ ^2^, ^3^ ^]^ The dissociation constant (*K*_d_) is a standard parameter that quantifies the binding affinity between interacting proteins. In vitro measurements of *K*_d_ under diluted conditions compared to the complex environment of living cells have been achieved using various techniques, such as surface plasmon resonance, fluorescence resonance energy transfer (FRET), and isothermal titration calorimetry (ITC).^[^ ^4^ ^]^ However, the need to extend these investigations to living organisms is becoming apparent, as the intracellular environment differs significantly from in vitro conditions. Factors such as the highly crowded and heterogeneous nature of the cellular milieu, the presence of nonspecific weak interactions with other macromolecules, and dynamic cellular processes introduce challenges not encountered in vitro.^[^ ^5^, ^6^ ^]^ The potency of PPIs can vary significantly depending on the extent of crowding, even within in vitro environments, underscoring the importance of directly measuring these interactions within living cells.^[^ ^7^ ^]^

Several methods have been developed for observing PPIs in living cells. The yeast two‐hybrid assay (Y2H) has been widely used for detecting PPIs because of its simplicity, although Y2H often suffers from false‐positive detection.^[^ ^8^ ^]^ Fluorescence‐based techniques, such as FRET, bioluminescence resonance energy transfer, and protein‐fragment complementation assays, have also been widely applied for PPI detection in living cells.^[^ ^9^, ^10^ ^]^ Recently, in‐cell NMR using isotope‐labeled proteins has been developed for observing protein interactions.^[^ ^5^, ^11^ ^]^ Despite the growing importance of directly probing PPIs in living cells, the lack of suitable methods for assessing quantitative *K*_d_ values in live cells remains a significant barrier to progress in this field. Among the available techniques, FRET has been used as a promising tool for quantitatively measuring *K*_d_ in living cells, mostly in eukaryotic cells.^[^ ^12^ ^]^ In addition to PPI measurements, quantitative FRET efficiency measurements, such as corrected FRET,^[^ ^13^ ^]^ FRETN,^[^ ^14^ ^]^ N_FRET_,^[^ ^15^ ^]^ and QuanTI‐FRET,^[^ ^16^ ^]^ have been applied in eukaryotic cells.

However, FRET approaches for quantitative PPI measurements, as well as quantitative FRET efficiency measurements, in living bacterial cells are rare and less successful.^[^ ^17^ ^]^ High autofluorescence backgrounds and more condensed conditions may be attributed to weak FRET signals in bacterial cells, resulting in a significant increase in false‐positive interactions.^[^ ^17^ ^]^ Previous studies assessed *K*_d_ within *E. coli* by measuring FRET signals collectively from heterogeneous cells, followed by disrupting cells using sonication and subsequent protein concentration measurement in vitro.^[^ ^18^ ^]^ FRET efficiency has also been employed as an indicator of PPI.^[^ ^19^ ^]^ However, FRET efficiency is dependent on the distance between two fluorescent proteins (FPs) and the concentrations of binder proteins, which limits its use as an indicator of binding affinity. As a result, it is highly demanding to develop a method for directly analyzing PPIs in living bacterial cells.

Here, we report a FRET‐based method, termed KD‐FRET, for quantitative PPI measurements in living *E. coli* cells, utilizing accurate FRET analysis. One fundamental limitation of using FRET for PPI measurements in bacterial cells is the weak FRET signals, which are often indistinguishable from background signals,^[^ ^17^ ^]^ leading to false‐positives. The strength of quantitative FRET measurements in in vitro single‐molecule assays lies in their ability to distinguish weak FRET signals from background signals.^[^ ^20^, ^21^ ^]^ We adapted the in vitro single‐molecule approach for use in live bacterial cells, enabling accurate correction of background noise and cross‐talks between donor and acceptor fluorophores. This approach not only improved the reliability of FRET measurements in bacterial systems but also allowed for the quantification of *K*_d_. We found that the direct excitation of the acceptor fluorophore is the major cause of false‐positives in PPI measurements using FRET. Interestingly, the FRET efficiency of the standard sample was similar both in vitro and in living *E. coli* cells. Unlike previous approaches, KD‐FRET allows the accurate determination of *K*_d_ values in living cells, including PPI pairs that are unstable or undetectable under standard in vitro conditions. Furthermore, we demonstrated the application of KD‐FRET in synthetic biology by increasing naringenin production in *E. coli* and lycopene production in *S. cerevisiae* through the engineering of biosynthetic pathway using interaction protein pairs, as confirmed by our method. These findings underscore the potential of KD‐FRET as a powerful tool for studying PPIs in their native cellular context and offering significant implications for both molecular biology and biotechnological applications.

## 2 Results and Discussion

### 2.1 Construction of Fluorescent Protein FRET Pairs for *K*_d_ Measurement

**Figure 1:**
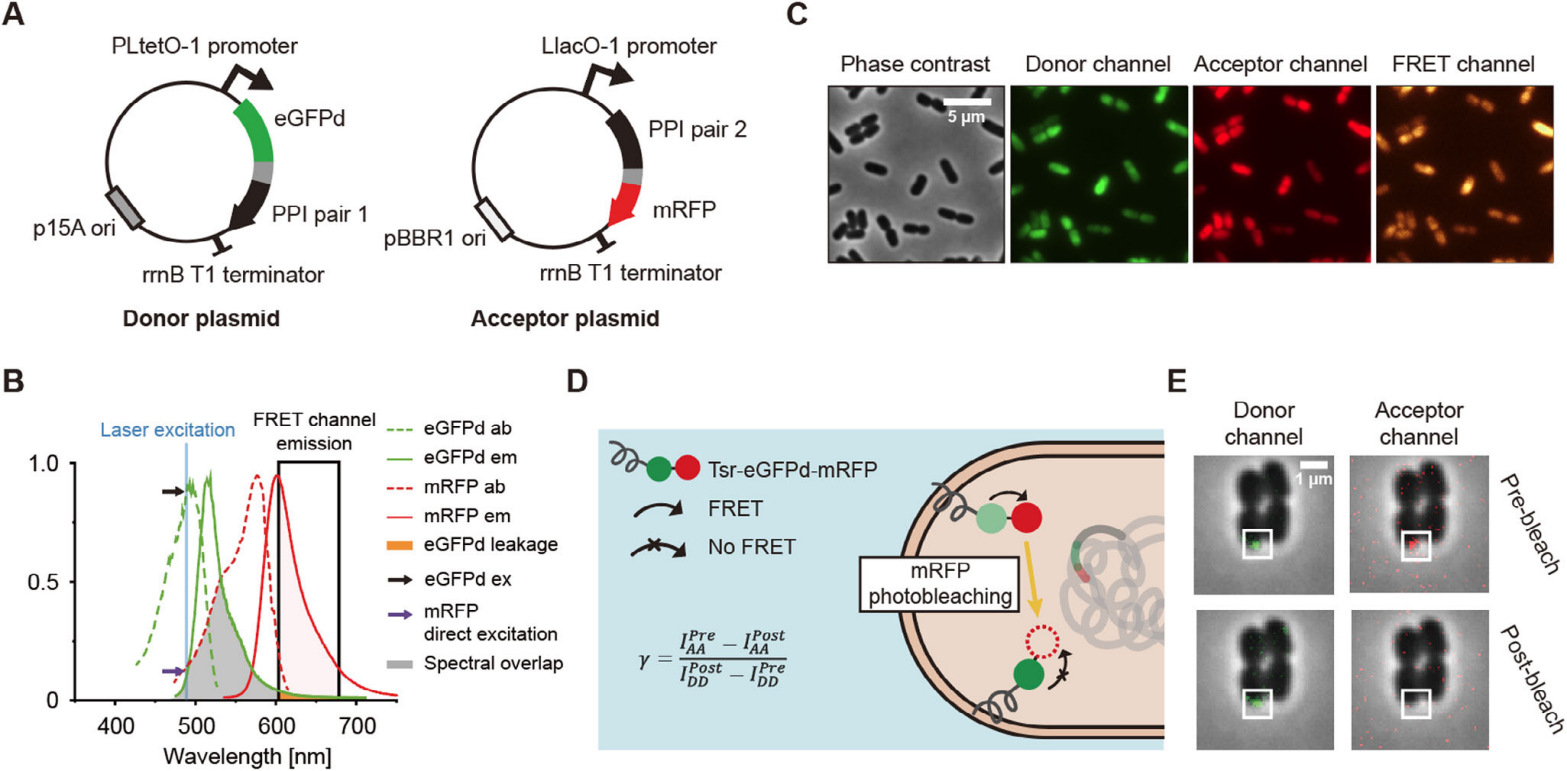
Schematic representation of the KD‐FRET system in *E. coli*. A) Schematic of the two‐vector system used for the concurrent expression of FRET donor and acceptor proteins. The donor plasmid expresses eGFPd fused to PPI pair 1, while the acceptor plasmid expresses mRFP fused to PPI pair 2. This system allows for independent and regulated expression of both fluorescent proteins in *E. coli* cells. B) Excitation and emission spectra of eGFPd and mRFP, the fluorescent proteins used in KD‐FRET experiments. The green and red dashed lines represent the excitation spectra of eGFPd and mRFP, respectively, while the solid green and red lines represent their corresponding emission spectra. The blue line indicates the donor excitation wavelength (488 nm), and the black arrow represents the eGFPd excitation at this wavelength. The highlighted gray area indicates the spectral overlap between eGFPd emission and mRFP absorption, which facilitates energy transfer. The orange area represents the leakage of eGFPd emission into the FRET channel. The purple arrow indicates the direct excitation of mRFP by the 488 nm laser. C) Representative images from the three‐channel FRET measurement system, showing *E. coli* cells imaged with phase contrast illumination, the donor channel (eGFPd), the acceptor channel (mRFP), and the FRET channel. Cells were imaged at 37 °C. The scale bar represents 5 µm. D) Schematic illustration of the Tsr‐eGFPd‐mRFP system used for calculating the γ factor. Prior to mRFP photobleaching, the eGFPd intensity is reduced due to FRET. After mRFP photobleaching, the eGFPd intensity increases as FRET is abolished. The γ factor is calculated by dividing the difference in acceptor intensity by the difference in donor intensity before and after mRFP photobleaching. E) Image of a fluorescent spot within a Tsr‐eGFPd‐mRFP expressing *E. coli* cell. The intensity of the fluorescent spot observed in the donor channel increases following mRFP photobleaching. The scale bar represents 1 µm.

FP‐fused PPI pair and their expression system are required to utilize FRET. The use of a single vector expressing the two fusion proteins for FRET‐induced abnormal cellular growth. Thus, we chose two compatible vectors with different induction systems and antibiotic resistance genes.^[^ ^22^ ^]^ In this study, pFF838, a chloramphenicol‐resistant tetracycline‐inducible vector, and pBBR6k, an ampicillin‐resistant IPTG‐inducible vector, were chosen as the donor and acceptor vectors, respectively (**Figure** **1**A; Tables S1 and S2, Supporting Information). For FPs, a substantial overlap between the donor emission and acceptor excitation spectra increases the probability of energy transfer, thereby enhancing FRET efficiency (Figure 1B). However, this overlap also introduces spectral crosstalk, including leakage of donor emission into the acceptor detection channel and direct excitation of the acceptor by the donor excitation laser. These crosstalk effects can lead to false‐positive FRET signals (Figure S1, Supporting Information). Therefore, the strength of crosstalk and bleed‐through, maturation time, and FRET efficiency were considered (Figure 1B; Figures S1 and S2, Supporting Information). eGFP and mRFP were initially chosen as the FRET pair to minimize the crosstalk between FPs while maintaining considerable FRET efficiency. However, the eGFP‐mRFP fused with a (GGGS)_2_ linker yielded very low FRET efficiency. Thus, we used eGFPd, an eGFP C‐terminal deletion mutant,^[^ ^23^ ^]^ as the donor FP, which significantly increased FRET efficiency between eGFPd and mRFP (Figure S2C, Supporting Information). The excitation and emission maxima of eGFPd were at 486 and 512 nm, respectively, which were almost identical to those of eGFP (Figure S3, Supporting Information). eGFPd‐(GGGS)_2_‐mRFP was used as a positive control for the high‐FRET pair. Donor and acceptor expression vectors were transformed into *E. coli* BW25993 cells.

### 2.2 Quantification of the Intracellular Protein Concentration

Quantification of cellular protein concentrations is essential for measuring the *K*_d_ of PPIs. Traditionally, inferring the concentration of intracellular proteins has relied on the process of cell lysis.^[^ ^5^, ^18^, ^24^ ^]^ However, we utilized the fusion of a protein of interest with a fluorescent reporter and leveraged imaging techniques to quantitatively determine both the number of proteins expressed and the volume of each cell (Figures S4 and S5, Supporting Information). We fused eGFPd or mRFP to the C‐terminus of Tsr, a membrane protein transported to the inner membrane, and the fused gene was inserted in place of the native *lacZ* gene. After consecutive imaging, the fluorescent spots were photobleached sequentially. The intensities of single eGFPd and mRFP spots were obtained by subtracting the spot intensities before and after bleaching (Figure S4, Supporting Information).^[^ ^25^ ^]^ The number of FPs in each cell was determined by dividing the total FP intensity by the intensity of a single FP. Then, the protein concentration of each cell was determined by the number of FP‐fused protein divided by the cellular volume which was in turn calculated from the two‐dimensional (2D) phase‐contrast image of each cell.

### 2.3 Accurate FRET Analysis of KD‐FRET for Living Bacterial Cells

The FRET efficiency can be measured using various techniques, such as sensitized emission, lifetime measurements, and acceptor photobleaching.^[^ ^26^ ^]^ The accurate FRET measurement in single‐molecule FRET has been developed based on sensitized emission FRET, the format of which is commonly available in most fluorescence microscopes equipped in cell biology laboratories.^[^ ^27^ ^]^ Sensitized emission FRET measurements, also known as three‐cube FRET or three‐filter FRET,^[^ ^28^ ^]^ consists of three detection channels: a donor channel (donor emission detection by donor excitation, *I*_DD_), an acceptor channel (acceptor emission detection by acceptor emission, *I*_AA_), and a FRET channel (acceptor emission detection upon donor excitation, *I*_DA_) (Figure 1C). FRET efficiency (*E*) is calculated as follows:

(1) $$E\;=\frac{I_{\mathrm{DA}}}{I_{\mathrm{DA}}+I_{\mathrm{DD}}}$$

The backgrounds and noise signals must be properly corrected from *I*_DD_, *I*_AA_, and *I*_DA_ to obtain accurate FRET efficiency.

(Step 1) Subtraction of image background signals. Image background signals (hereafter “BG”) should be removed. We measured the intensity of each image frame, i.e., $I_{DD}^{BG},\;I_{AA}^{BG},\;\mathrm{and}\;I_{DA}^{BG}$.

(Step 2) Subtraction of cellular autofluorescence. Bacterial cells have autofluorescence. Wild‐type BW25993 cells were used for autofluorescence correction of each channel.

(Step 3) Leakage correction. The use of two different FPs for FRET measurements inevitably resulted in spectral crosstalk. Specifically, leakage (Lk) refers to the unintended detection of donor fluorescence by the acceptor channel, while direct excitation refers to the excitation of the acceptor fluorophore directly by donor excitation. Leakage or direct excitation can generate false FRET signals. Therefore, correction of these two factors is important for obtaining appropriate FRET signals. The contribution of Lk can be determined using *E. coli* cells transformed with plasmids expressing donor proteins (hereafter “Donly”) by dividing the FRET channel intensity ($I_{DA}^{Donly}$) by the donor channel intensity ($I_{DD}^{Donly}$).

(Step 4) Direct excitation correction. The direct excitation (Dir) can be corrected by using *E. coli* cells expressing acceptor proteins (hereafter “Aonly”): the FRET channel intensity ($I_{DA}^{Aonly}$) was divided by the acceptor channel intensity ($I_{AA}^{Aonly}$).

The FRET intensity of the FRET sample ($I_{DA}^{FRET}$) was then corrected by eliminating leakage and direct excitation using the following equation:

(2) $$Corrected\;FRET,\;F^{FRET}=I_{DA}^{FRET}-\left(Lk*I_{DD}^{FRET}\right)-\left(Dir*I_{AA}^{FRET}\right)$$

(Step 5) Gamma (γ) factor correction. The γ factor, also known as the detection efficiency correction factor, accounts for the difference in detection efficiency between the donor and acceptor channels. The γ factor can be obtained by measuring the intensity changes of the immobilized single donor–acceptor fusion protein before and after acceptor photobleaching, $\;\gamma =\frac{\Delta I_{AA}}{\Delta I_{DD}}$ (Figure 1D).^[^ ^29^ ^]^ To obtain γ factor using the microscope, the membrane protein Tsr‐fused eGFPd‐mRFP tandem protein was expressed from chromosomally integrated *tsr‐eGFPd‐mRFP* gene (SY004 strain). The change in the intensity of a membrane‐localized eGFPd‐mRFP spot upon mRFP photobleaching was measured in both donor and acceptor channels. The spot intensity of the donor channel increased after mRFP was photobleached, owing to the destruction of the FRET acceptor (Figure 1D,E). The distribution of γ factor was obtained from the intensities of the eGFPd‐mRFP spot before (*pre*) and after (*post*) mRFP photobleaching (Figure 1D). The γ factor was measured to be 0.45 (*n* = 31) by fitting the distribution to a Gaussian function.

Finally, FRET efficiency (*E*) was calculated using the following equation:

(3) $$E=\frac{F^{FRET}}{F^{FRET}+\gamma *I_{DD}^{FRET}}$$

(Step 6) Outlier removal. The emergence of outliers with either negative or extremely large FRET efficiency values is inevitable due to a series of noise correction procedures.^[^ ^30^ ^]^ Therefore, it is often required to establish a threshold for outlier removal. Given that outliers are frequently associated with low intracellular donor concentrations (weak donor signals), we used a threshold based on the mean donor channel intensity subtracted by its standard deviation.^[^ ^31^ ^]^ Cells exhibiting donor channel intensities below this threshold were considered outliers and excluded from the analysis.

(Step 7) Dissociation constants. After applying all the correction procedures, individual cells were binned according to their intracellular acceptor concentrations. The donor concentration and FRET efficiency in each bin were averaged and plotted. Consequently, the data were fitted using a quadratic binding equation to determine the *K*_d_ values (Figure S6, Supporting Information).^[^ ^32^ ^]^ The plots fitted using the quadratic binding equation are shown as 2D plots instead of 3D plots for simplicity. The overall workflow is summarized in Figure S7 (Supporting Information).

### 2.4 Validation of KD‐FRET Using the Positive and Negative Controls in Living Bacterial Cells

Each correction step was then applied to the positive and negative controls of the FRET pairs to determine *K*_d_. As a negative control, we expressed both eGFPd and mRFP using two different vectors, respectively. The negative control should result in a FRET efficiency value close to zero after full correction, and its *K*_d_ should not be measured because there is no interaction between the FPs. The directly fused eGFPd and mRFP (eGFPd‐(GGGS)_2_‐mRFP) was used as a positive control, with its FRET efficiency expected to be independent of eGFPd‐mRFP concentration. *K*_d_ cannot be determined for eGFPd‐mRFP, as it represents a case of an infinite association constant.

**Figure 2:**
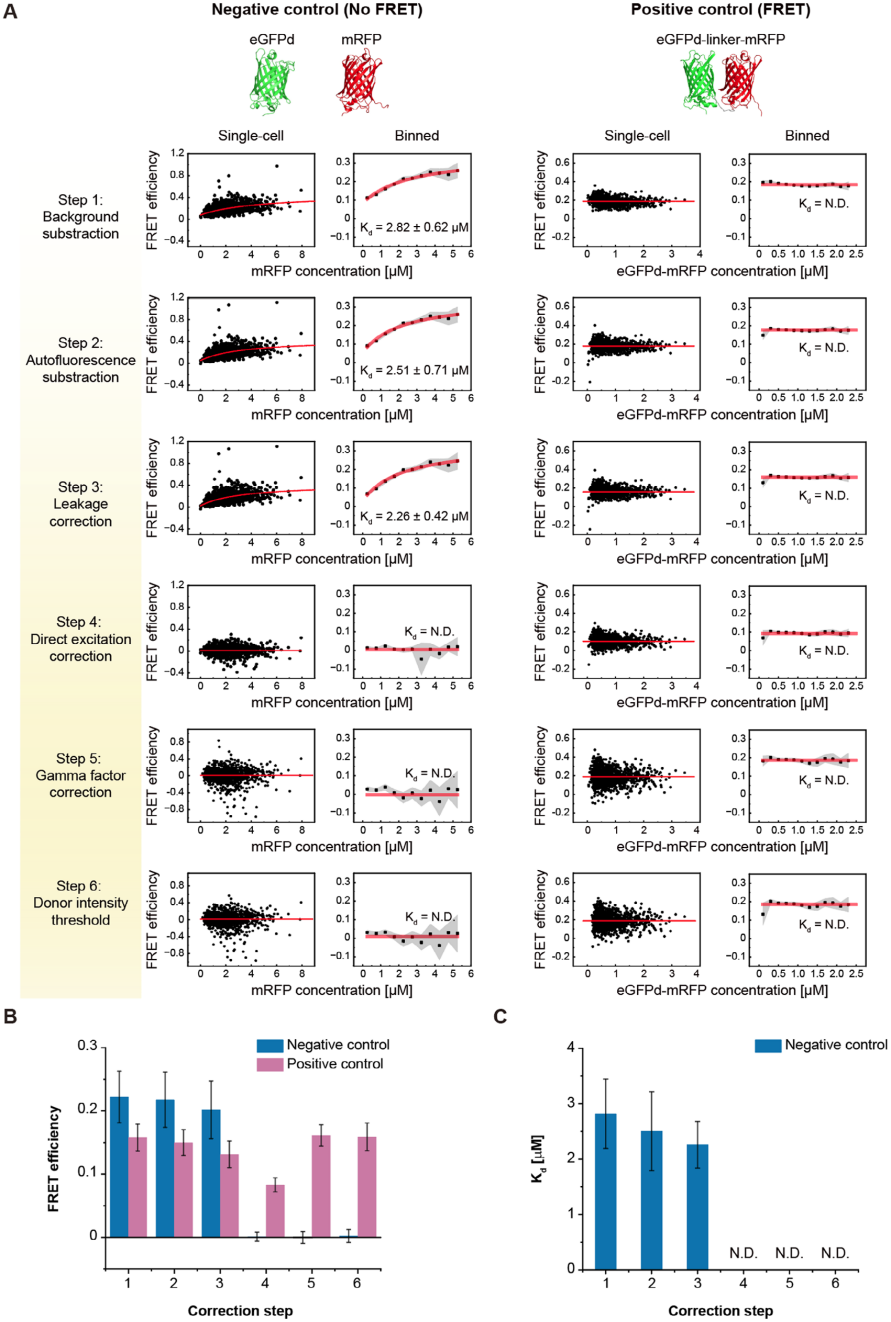
Stepwise correction of FRET efficiency and its impact on K_d_ measurements in *E. coli* cells. A) Data analysis processes and corresponding single‐cell and binned scatter plots for negative (left) and positive (right) controls. The plots depict the step‐by‐step correction of FRET efficiency, including background subtraction, autofluorescence subtraction, leakage correction, direct excitation correction, γ factor correction, and donor intensity thresholding. In the negative control, incomplete corrections resulted in scatter plots displaying a binding curve shape, leading to false‐positive *K*_d_ values. In the positive control, the absence of γ factor correction led to an underestimation of FRET efficiency compared to in vitro FRET measurements of the purified positive control. *K*_d_ values are reported as mean ± standard deviation (SD) of triplicates. The gray‐shaded regions represent the 95% confidence intervals for the binned mean FRET efficiency. B) Analysis of FRET efficiency values and C) *K*_d_ for the negative (blue) and positive (red) controls across the correction steps. The data highlight how each correction step affects the accuracy of the *K*_d_ and FRET efficiency measurements, emphasizing the importance of correction processes for reliable in‐cell FRET analysis. Error bars were obtained from the SDs of three independent measurements. N.D.: Not determined.

**Figure** **2**A shows the FRET efficiency and *K*_d_ measurements at each correction step for negative controls. Notably, after step 1 correction, false interactions were observed with *K*_d_ = 2.82 ± 0.62 × 10^−6^ m. The FRET efficiency, indicating the interaction between eGFPd and mRFP, initially increased as the concentration of the acceptor protein (mRFP) increased within the cell, yielding a positive *E* value and pseudo‐*K*_d_ (Figure 2B,C). Until step 3 of the leakage correction, the false‐positive *K*_d_ was measured to be 2.26 ± 0.42 × 10^−6^ m. Interestingly, however, after step 4 direct excitation correction, a false‐positive *K*_d_ was not detected. The FRET efficiency was also close to zero after step 4 correction. These results indicate that the false‐positive result for protein interaction using FRET pairs in living bacterial cells mostly comes from the crosstalk of direct excitation. To evaluate the effect of direction excitation correction, we applied it in step 3, followed by leakage correction in step 4 (Figure S8, Supporting Information). Again, the direct excitation correction removed the false‐positive *K*_d_ value. These results clearly demonstrate that the false‐positive *K*_d_ measurement occurs in living bacterial cells due to the spectral crosstalk. It is essential to perform direction excitation correction, even for fast qualitative screening for PPI pairs in living *E. coli* using FRET, to remove the false‐positive results. We further validated the importance of direct excitation correction using another non‐interacting pair (eGFPd and ^N^DD_MlnC_‐mRFP), which similarly showed elimination of false‐positive signals after proper correction (Figure S9, Supporting Information). Meanwhile, γ factor correction and outlier removal are not essential for *K*_d_ measurement using FRET.

The results of the positive FRET control are presented in Figure 2B,C. As predicted, the FRET efficiency was independent of the FP concentration and *K*_d_ was not determined. After full correction to step 6, the FRET efficiency was measured to be 0.16 ± 0.02 for eGFP‐mRFP in living *E. coli*. The results of the positive control also support the robustness of our correction processes.

### 2.5 Comparison of the FRET Efficiencies Measured In Vitro and in Living Cells

It has been an interesting question whether the FRET efficiencies measured in vitro matches those measured in living cells.^[^ ^33^ ^]^ Therefore, we compared the FRET efficiency of the positive control measured in living cells to that measured in vitro. To determine the FRET efficiency in vitro, we used the sensitized‐emission FRET measurement with correction of all crosstalks.^[^ ^20^, ^34^ ^]^ We determined the extinction coefficients of the purified free eGFPd and free mRFP at the identical wavelength to KD‐FRET measurement (488 nm for eGFPd excitation, 580 nm for mRFP excitation), which were 58 705.8 m ^−1^ cm^−1^ ($\varepsilon_{488\;nm}^{eGFPd}$) and 53 000 m ^−1^ cm^−1^ ($\varepsilon_{580\;nm}^{mRFP}$), respectively. As a result, the in vitro FRET efficiency of the positive control was measured to be 0.20 ± 0.03 by using a fluorometer, which is in a similar range as the FRET efficiency of living cells (0.16 ± 0.02). These results indicate that FRET efficiency quantitatively measured in vitro closely mirrors that observed in living *E. coli* cells. This comparative result further supports the robustness of our analytical approach for determining intracellular FRET efficiency.

### 2.6 Dissociation Constant Measurements of Known Interaction Pairs by KD‐FRET

**Figure 3:**
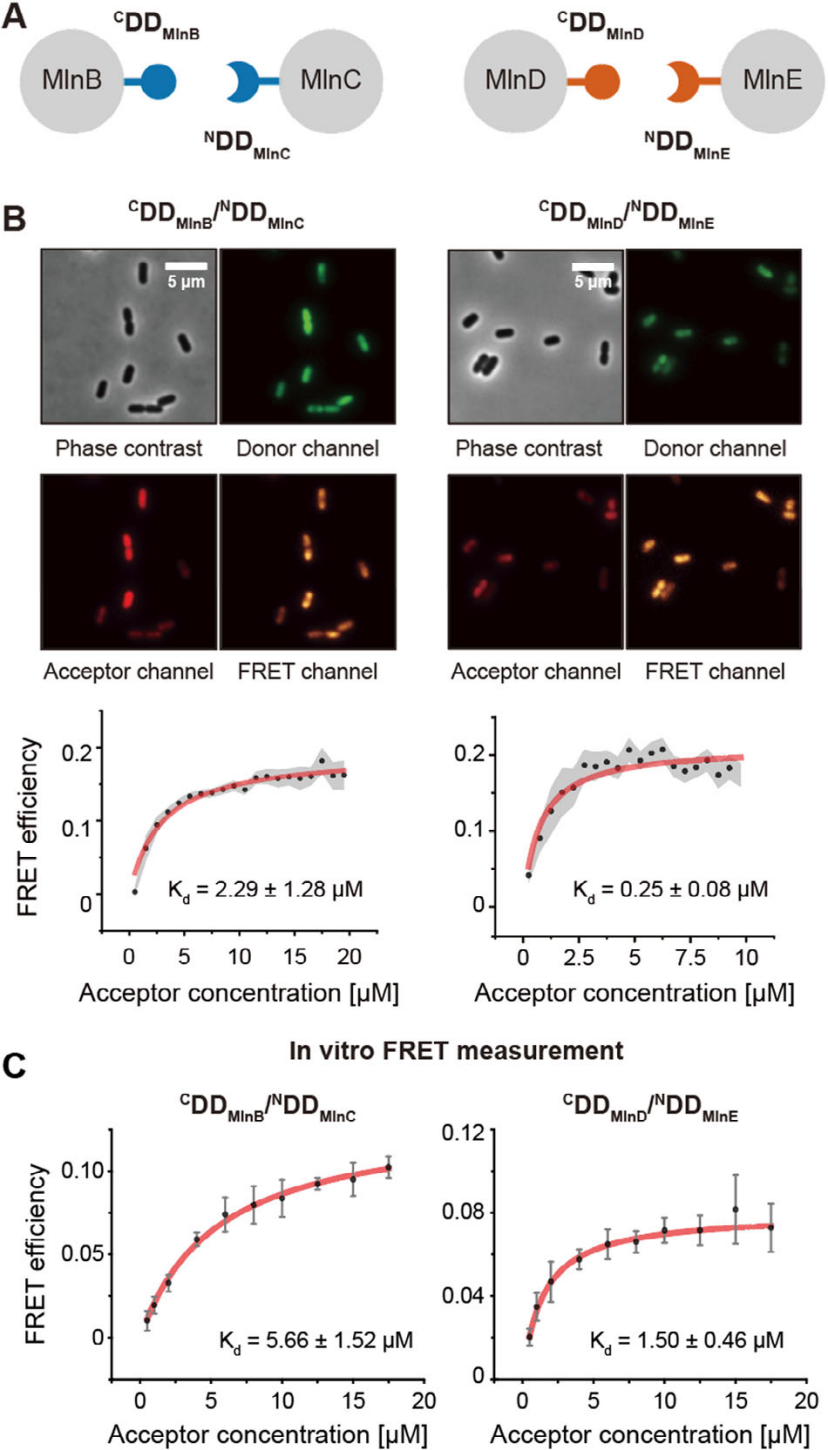
*K*_d_ determinations in living cells and in vitro using accurate FRET measurements. A) Schematic representation of the interactions between docking domains (DDs) from the macrolactin trans‐AT polyketide synthases (PKSs) MlnB/MlnC and MlnD/MlnE. The C‐terminal DDs (^C^DD) and N‐terminal DDs (^N^DD) from MlnB–MlnC and MlnD–MlnE specifically interact with their corresponding partners, forming well‐defined protein–protein interaction (PPI) pairs. B) Representative in‐cell *K*_d_ measurements of ^C^DD_MlnB_/^N^DD_MlnC_ (left) and ^C^DD_MlnD_/^N^DD_MlnE_ (right) in living *E. coli* cells using KD‐FRET. Fluorescence microscopy images show cells in phase contrast, donor channel (eGFPd), acceptor channel (mRFP), and FRET channel. The corresponding FRET efficiency versus acceptor concentration plots are presented below, with *K*_d_ values determined to be 2.29 ± 1.28 × 10^−6^ m for ^C^DD_MlnB_/^N^DD_MlnC_ and 0.25 ± 0.08 × 10^−6^ m for ^C^DD_MlnD_/^N^DD_MlnE_ (mean ± SD). Measurements were performed in triplicate at 37 °C. The gray‐shaded regions represent the 95% confidence intervals for the binned mean FRET efficiency. The scale bar represents 5 µm. C) Representative in vitro *K*_d_ measurements of the same PPI pairs, ^C^DD_MlnB_/^N^DD_MlnC_ (left) and ^C^DD_MlnD_/^N^DD_MlnE_ (right), conducted with purified proteins in PBS buffer using a plate reader. The in vitro *K*_d_ values were measured to be 5.66 ± 1.52 × 10^−6^ m for ^C^DD_MlnB_/^N^DD_MlnC_ and 1.50 ± 0.46 × 10^−6^ m for ^C^DD_MlnD_/^N^DD_MlnE_ (mean ± SD). Measurements were performed in triplicate at 37 °C.

Then, to confirm the robustness of KD‐FRET, we employed a well‐characterized PPI system consisting of docking domains (DDs) from polyketide synthases (PKS). DDs of PKSs are positioned at the C‐ and N‐termini of PKSs, interacting exclusively with their designated pairs and ensuring the organization of the multienzyme complex.^[^ ^21^, ^22^ ^]^ We chose the C‐terminal DD (^C^DD_MlnB_) and N‐terminal DD (^N^DD_MlnC_) derived from macrolactin *trans*‐AT PKS, MlnB/MlnC and MlnD/MlnE, whose interaction is characterized by the formation of a four‐helix bundle, with complementary branched‐chain amino acids at the core interface mediating specific recognition and stabilizing the complex assembly (**Figure** **3**A).^[^ ^35^ ^]^ The *K*_d_ values, reported as 2.9 ± 1.1 × 10^−6^ and 4.8 ± 2.3 × 10^−6^ m, were measured in vitro using FRET without applying crosstalk corrections.^[^ ^36^ ^]^

We directly measured the *K*_d_ values of ^C^DD_MlnB_/^N^DD_MlnC_ and ^C^DD_MlnD_/^N^DD_MlnE_ pairs within the cellular context by utilizing our FRET measurement system (Figure 3B). ^C^DD_MlnB_ and ^C^DD_MlnD_ were labeled with eGFPd, whereas ^N^DD_MlnC_ and ^N^DD_MlnE_ were labeled with mRFP. The FP‐labeled ^C^DD_MlnB_/^N^DD_MlnC_ and ^C^DD_MlnD_/^N^DD_MlnE_ pairs were expressed using two vector systems (Figure 1A). We applied the correction until step 6 for *K*_d_ determination. The *K*_d_ values for ^C^DD_MlnB_/^N^DD_MlnC_ and ^C^DD_MlnD_/^N^DD_MlnE_ pairs in living *E. coli* were measured to be 2.29 ± 1.28 × 10^−6^ and 0.25 ± 0.08 × 10^−6^ m, respectively (Figure 3B). To confirm whether the measured *K*_d_ values were reasonable, we purified the identical fusion proteins and measured their *K*_d_ values using FRET in vitro. The *K*_d_ values for ^C^DD_MlnB_/^N^DD_MlnC_ and ^C^DD_MlnD_/^N^DD_MlnE_ pairs were 5.66 ± 1.52 × 10^−6^ and 1.50 ± 0.46 × 10^−6^ m, respectively (Figure 3C). Furthermore, we measured the K_d_ value of ^C^DD_MlnB_/^N^DD_MlnC_ pair using ITC, which yielded a value of 6.85 ± 0.41 × 10^−6^ m (Figure S10, Supporting Information). The in‐cell *K*_d_ values obtained through KD‐FRET measurements closely matched those obtained through in vitro measurements, providing additional support for the reliability of our methodology. The difference in *K*_d_ values between in vitro and in‐cell assays may be due to the complex nature of the intracellular environment. Factors such as macromolecular crowding, increased viscosity, nonspecific interactions with other macromolecules, and enhanced protein stability in the cellular milieu may collectively contribute to the observed differences, resulting in lower *K*_d_ values in cells.^[^ ^5^, ^37^, ^38^ ^]^

### 2.7 *K*_d_ of UvrA/UvrB Interacting Pair in living *E. Coli* Cells

**Figure 4:**
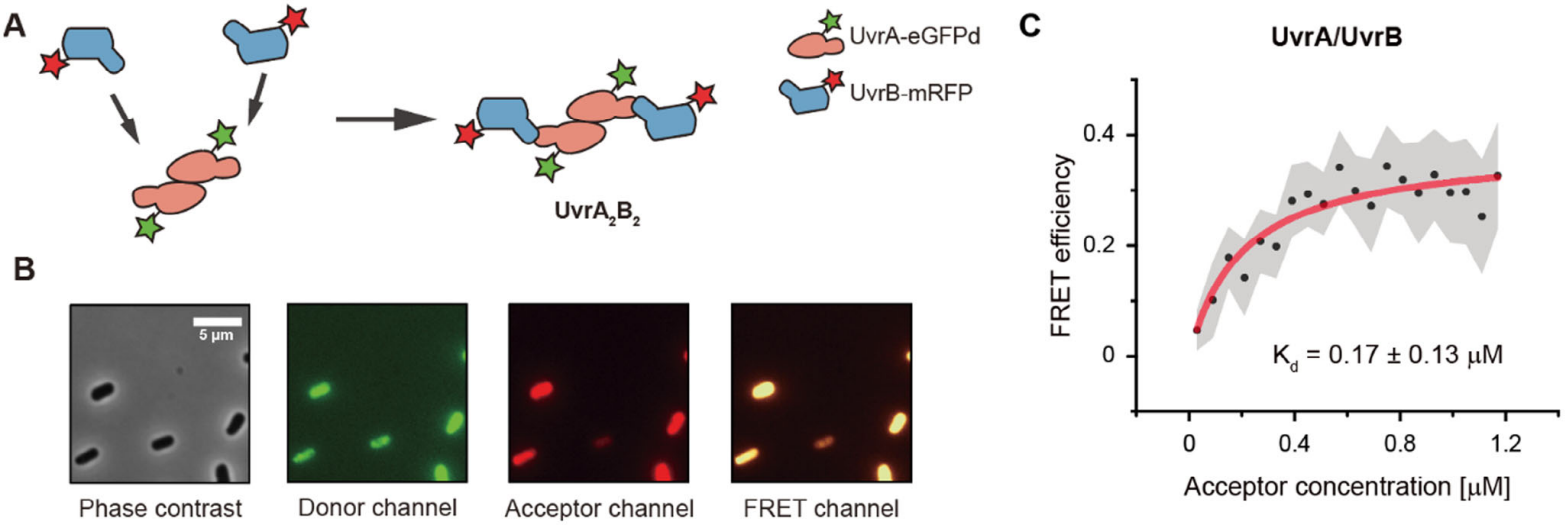
In‐cell *K*_d_ determination of the UvrA/UvrB interaction using KD‐FRET in living *E. coli*. A) Schematic representation of the UvrA/UvrB protein–protein interaction. UvrA is labeled with eGFPd, and UvrB is labeled with mRFP. These proteins interact in a 1:1 stoichiometry to form the UvrA_2_B_2_ complex. B) Fluorescence microscopy images showing the expression of UvrA‐eGFPd and UvrB‐mRFP in the *E. coli* BW25993 strain, which has a double deletion of the uvrA and uvrB genes. The scale bar represents 5 µm. C) Representative in‐cell *K*_d_ measurement for the UvrA/UvrB interaction performed at 37 °C. The FRET efficiency is plotted against acceptor concentration, and the *K*_d_ value was determined to be 0.17 ± 0.13 × 10^−6^ m from three independent measurements (mean ± SD). The gray‐shaded regions represent the 95% confidence intervals for the binned mean FRET efficiency.

To broaden the spectrum of protein–protein interaction pairs amenable to *K*_d_ measurements using our approach, we measured the in‐cell *K*_d_ of the UvrA/UvrB protein‐protein interaction pair, a relatively large protein complex related to nucleotide excision repair within *E. coli*. UvrA and UvrB proteins interact with each other in a 1:1 ratio to form the UvrA_2_B_2_ complex having in vitro *K*_d_ ≈ 0.8 × 10^−6^ m determined by isothermal titration calorimetry (**Figure** **4**A).^[^ ^39^ ^]^ The expression of UvrA/UvrB pair was prepared using the two‐vector system (Figure 1A), and the vectors were transformed into the UvrA/UvrB knockout BW25993 cells. We also performed the correction until step 6 to determine *K*_d_. As a result, in cell *K*_d_ of UvrA/UvrB pair was measured as 0.17 ± 0.13 × 10^−6^ m (Figure 4B,C). These data show that our method can be applied not only to small heterologous protein pairs, but also to large PPI pairs in *E. coli*.

### 2.8 Measurement of *K*_d_ Values of PPI Pairs That are Unmeasurable In Vitro

**Figure 5:**
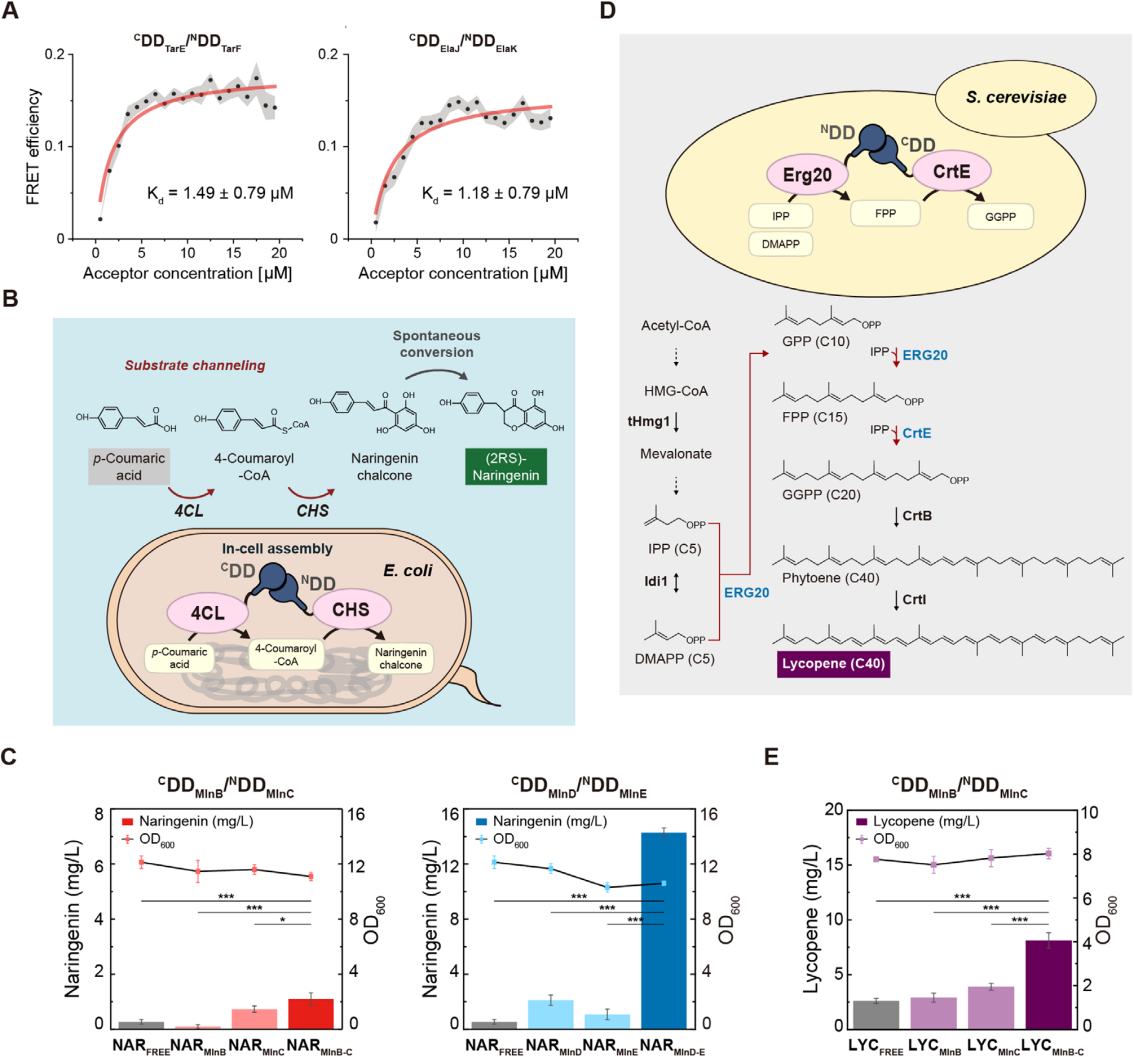
Applications of in‐cell *K*_d_ measurements using accurate FRET analysis for synthetic biology. A) Representative in‐cell *K*_d_ measurements of DD pairs, ^C^DD_TarE_/^N^DD_TarF_ and ^C^DD_ElaJ_/^N^DD_ElaK_. The *K*_d_ values were measured to be 1.49 ± 0.79 × 10^−6^ m for ^C^DD_TarE_/^N^DD_TarF_ and 1.18 ± 0.79 × 10^−6^ m for ^C^DD_ElaJ_/^N^DD_ElaK_ using KD‐FRET in living *E. coli* cells (mean ± SD). Measurements were performed in triplicate at 37 °C. The gray‐shaded regions represent the 95% confidence intervals for the binned mean FRET efficiency. B) Schematic representation of the experimental design for the artificial metabolon system utilizing the enzymes 4‐coumaroyl‐CoA ligase (4CL) and chalcone synthase (CHS). In this system, *p*‐coumaric acid is converted into 4‐coumaroyl‐CoA by 4CL, followed by the conversion of 4‐coumaroyl‐CoA into naringenin chalcone by CHS. Finally, naringenin chalcone spontaneously forms (2RS)‐naringenin. The metabolon system was constructed by fusing 4CL and CHS with either ^C^DD_MlnB_/^N^DD_MlnC_ or ^C^DD_MlnD_/^N^DD_MlnE_ pairs, facilitating substrate channeling within *E. coli* cells. C) Naringenin production levels (left *y*‐axis, bar graphs) in the artificial metabolon system using the ^C^DD_MlnB_/^N^DD_MlnC_ pair (left) and the ^C^DD_MlnD_/^N^DD_MlnE_ pair (right). The OD_600_ values of *E. coli* cultures are shown as line graphs (right *y*‐axis). The metabolon system utilizing the ^C^DD_MlnD_/^N^DD_MlnE_ pair, which exhibited a lower *K*_d_ in living cells, resulted in a more significant increase in naringenin production compared to the system using the ^C^DD_MlnB_/^N^DD_MlnC_ pair. Measurements were performed in triplicate at 30 °C and error bars reflect standard deviation. Statistical significance was determined using one‐way ANOVA followed by Tukey's post‐hoc test (***p < 0.001, *p < 0.05). D) Schematic representation of the experimental design for the artificial metabolon system utilizing the enzymes farnesyl pyrophosphate synthase (FPP synthase, ERG20) and geranylgeranyl pyrophosphate synthase (GGP synthase, CrtE). In this system, ERG20 catalyzes the sequential condensation of isopentenyl pyrophosphate (IPP, C5) with dimethylallyl pyrophosphate (DMAPP, C5), and then with the resultant geranyl pyrophosphate (GPP, C10) to the ultimate product farnesyl pyrophosphate (FPP, C15). And a downstream enzyme CrtE synthesizes geranylgeranyl pyrophosphate (GGPP, C20) from C5‐IPP and C15‐FPP. ERG20 and CrtE were fused with ^C^DD_MlnB_/^N^DD_MlnC_ pair to assemble two enzymes for substrate channeling within *S. cerevisiae* cells. E) Lycopene production levels (left *y*‐axis, bar graphs) in the artificial metabolon system using the ^C^DD_MlnB_/^N^DD_MlnC_ pair. The metabolon system utilizing the ^C^DD_MlnB_/^N^DD_MlnC_ pair resulted in the 3.4‐fold increased lycopene production. Measurements were performed in triplicate at 30 °C and error bars reflect standard deviation. Statistical significance was determined using one‐way ANOVA followed by Tukey's post‐hoc test (***p < 0.001).

Given that PPIs are highly influenced by their surrounding environment, the affinity of these interactions may vary depending on the type of reaction buffer used or may not be measurable consistently in vitro.^[^ ^3^, ^40^ ^]^ For example, several studies have shown that ion concentration and the type of buffer used alter PPIs, even at physiological pH.^[^ ^41^ ^]^ In our study, the DD pairs of TarE/TarF and ElaJ/ElaK, known to interact,^[^ ^42^ ^]^ exhibited either unstable interactions or undetectable *K*_d_ values in vitro (Figure S11, Supporting Information). However, our approach directly measured the *K*_d_ values within living *E. coli* cells, i.e., 1.49 ± 0.79 × 10^−6^ and 1.18 ± 0.79 × 10^−6^ m for ^C^DD_TarE_–^N^DD_TarF_, ^C^DD_ElaJ_–^N^DD_ElaK_ pairs, respectively (**Figures** **5**A and S12, Supporting Information). KD‐FRET appears to provide a reliable assessment of binding affinities, free from potential artifacts due to the quality of the purified proteins and different buffer conditions, although further investigation is still required. This result further highlights the value of quantifying protein interaction strength within a cellular context.

### 2.9 Application of the PKS Interaction Pairs Analyzed in Living Cells for Synthetic Biology

A metabolon refers to a dynamic and intricately organized protein complex comprising enzymes involved in specific metabolic pathways.^[^ ^43^ ^]^ This molecular assembly enhances the efficiency of biochemical reactions within a given metabolic pathway. It facilitates the sequential conversion of metabolites by channeling intermediates from one enzyme to the next. Metabolic engineering, aimed at enhancing the efficient production of desired substances, could potentially benefit from emulating the concept of a metabolon. It may be possible to further increase the production yields by bringing enzymes into a closer proximity, similar to a metabolon.^[^ ^44^ ^]^

The biosynthesis of (2RS)‐naringenin, a predominant member and fundamental building block of diverse flavonoids, involves the enzymatic conversion of *p*‐coumaric acid to 4‐coumaroyl‐CoA by 4‐coumaroyl‐CoA ligase (4CL), followed by the formation of naringenin chalcone from 4‐coumaroyl‐CoA catalyzed by chalcone synthase (CHS). Naringenin chalcone undergoes a spontaneous conversion to (2RS)‐naringenin (Figure 5B).^[^ ^45^ ^]^ In the cellular context, these enzymes are independently expressed, existing without specific organization within the cell, and solely depending on diffusion for interaction. We used the ^C^DD_MlnB_/^N^DD_MlnC_ or ^C^DD_MlnD_/^N^DD_MlnE_, whose K_d_ values have been determined in living *E. coli* by our KD‐FRET method, to co‐localize the 4CL and CHS enzymes and improve biocatalytic efficiency of flavonoid production in *E. coli*. The ^C^DDs were tagged to the C terminus of 4CL, while the ^N^DDs were attached to the N terminus of CHS (Figure 5B), facilitating enzyme assembly via selective interactions between the cognate DD pairs. To evaluate the influence of 4CL and CHS on enzymatic activity and expression levels within cells when fused to DD pairs, control strains containing only a single DD tag were also constructed (Table S3, Supporting Information). The strain NAR_MlnB_ (harboring 4CL‐^C^DD_MlnB_) produced less naringenin than the strain NAR_Free_, which possesses untagged 4CL and CHS. In contrast, the strains NAR_MlnC_, NAR_MlnD_, and NAR_MlnE_ (containing ^N^DD_MlnC_‐CHS, 4CL‐^C^DD_MlnD_, and ^N^DD_MlnE_‐CHS, respectively) exhibited a modest enhancement in naringenin production relative to the strain NAR_Free_. In our previous study, we observed that DD tagging did not influence enzymatic activity of the 4CL or CHS.^[^ ^35^ ^]^ Therefore, the differences in production among the control strains are presumed to result from differences in the expression levels of the fusion proteins. As a result, the assembly strains (NAR_MlnB‐C_ and NAR_MlnD‐E_) produced higher amount of naringenin than the control strains, likely attributable to the influence of DD‐based substrate channeling. Interestingly, the assembly strain utilizing ^C^DD_MlnB_/^N^DD_MlnC_, exhibiting a *K*_d_ value of 2.29 ± 1.28 × 10^−6^ m, showed a 4.1‐fold increase in naringenin production (Figure 5C). Furthermore, another assembly strain utilizing ^C^DD_MlnD_/^N^DD_MlnE_, exhibiting a stronger interaction with a *K*_d_ of 0.25 ± 0.08 × 10^−6^ m, showed a remarkable 25.5‐fold increase in naringenin production compared to NAR_Free_ strain (Figure 5C). These results indicate that the biocatalytic efficiency of the artificial metabolon is enhanced when using PPI domain pairs with lower *K*_d_ values.

However, this approach may not be universally applicable across all metabolic pathways. To explore whether the PPI domain interactions observed in *E. coli* could be transferred to other host organisms, we applied the ^C^DD_MlnB_/^N^DD_MlnC_ pair within the lycopene biosynthetic pathway in genetically engineered *Saccharomyces cerevisiae*. The pathway was constructed by integrating the *tHMG1* and *crtI* genes (encoding hydroxymethylglutaryl‐CoA reductase and phytoene desaturase, respectively) onto the *S. cerevisiae* chromosome. In addition, the *erg20* gene (encoding farnesyl pyrophosphate synthase) and *crtE* gene (encoding geranylgeranyl pyrophosphate synthase) were co‐expressed along with the *crtB* gene (encoding phytoene synthase) on a replicative plasmid, resulting in the strain LYC_Free_ (Figure 5D and Table S3, Supporting Information). We selected ERG20 and CrtE as target enzymes to be assembled in a lycopene‐producing yeast host, anticipating that efficient substrate channeling among C5, C10, and C15 intermediates would be facilitated, leading to enhanced lycopene production. The ^C^DD_MlnB_ and ^N^DD_MlnC_ were fused to the N termini of CrtE and ERG20, respectively, resulting in strain LYC_MlnB‐C_, which comprises ^C^DD_MlnB_‐CrtE and ^N^DD_MlnC_‐ERG20. Control strains containing one of the tags with no assembly functionality were also constructed (Table S3, Supporting Information). Lycopene production in the assembly strain was 3.4‐fold higher than in the untagged control strain (Figure 5E). The DD‐tagging of either CrtE or ERG20 alone in the control strains did not significantly affect lycopene production. These results showed that the efficacy of the artificial metabolon was preserved when the identical PPI domain pair was utilized in metabolic pathways in different host organisms.

Our results demonstrate that utilizing KD‐FRET enables the screening of interactions in living cells more efficiently, providing a more streamlined approach for constructing artificial metabolons.

## 3 Discussion

Despite the importance of PPIs, direct methods to measure their strength in living *E. coli* cells have been lacking, largely due to high autofluorescence and the complexity of the cellular environment. Furthermore, it is known that intracellular FRET and NMR signals used to observe PPIs in *E. coli* typically exhibit broader distributions and lower signal‐to‐noise ratios compared to those in other organisms.^[^ ^17^, ^46^ ^]^ The dense intracellular environment of *E. coli* may contribute to the reduced diffusivity of proteins and variations in FRET efficiency, complicating quantitative *K*_d_ measurements.^[^ ^47^ ^]^ Although in vitro studies provide valuable insights, they often fail to replicate the intracellular milieu, highlighting the need for in‐cell approaches. Our work presents a FRET‐based method for quantitatively measuring *K*_d_ values of PPIs directly within living *E. coli* cells. This method overcomes previous challenges by eliminating the need for protein purification and allowing measurements in a natural cellular context. The use of correction factors in KD‐FRET significantly reduced false‐positives, enhancing the reliability of FRET efficiency measurements. This technique can address the instability of PPIs in in vitro conditions by measuring them directly within the cellular environment.

Compared to other methods for measuring PPIs in bacterial cells, KD‐FRET offers distinct advantages. The bacterial two‐hybrid (B2H) system detects PPIs qualitatively rather than quantitatively and often requires genetic modifications that may alter the native interaction environment.^[^ ^10^, ^48^ ^]^ In‐cell NMR provides atomic‐level resolution but has limited applicability due to complex sample preparation steps, such as isotope labeling and reintroducing labeled proteins into cells via electroporation, as well as the need for specialized NMR equipment.^[^ ^11^, ^46^, ^49^ ^]^ In contrast, KD‐FRET is more accessible, requiring only a conventional fluorescence microscope and routine cloning for sample preparation. Traditional FRET‐based approaches can detect PPIs but suffer from reduced sensitivity in bacterial cells due to background autofluorescence and broad/low FRET signals.^[^ ^17^ ^]^ KD‐FRET overcomes these limitations, enabling quantitative *K*_d_ measurements.

Minimizing autofluorescence inherent to growth media is crucial for detecting weak FRET signals. Rich media, such as Luria‐Bertani broth, produce a high autofluorescence background. Therefore, KD‐FRET experiments were conducted in minimal M9 media to reduce autofluorescence. In addition, a two‐vector system was employed to independently regulate donor and acceptor protein expression levels by varying inducer concentrations. These strategies allowed control of protein expression levels within the expected *K*_d_ range, facilitating quantitative FRET efficiency measurements from the weak FRET signal of *E. coli* cells. As described before, autofluorescence, leakage, direct excitation, and γ‐factor corrections were applied during every imaging session for each sample. These correction steps were conducted in the same environment as the measurements for the corresponding PPI pairs, ensuring that any potential effect from *E. coli* autofluorescence or environmental variables was effectively accounted for and removed.

In this study, we used only cytosolic proteins, which diffuse freely throughout the *E. coli* cell body. However, if the proteins of interest are membrane proteins that are localized to the cell membrane, the calculation of intracellular concentration becomes more complex, as the system shifts from a 3D to a 2D framework. In such cases, an additional 2D definition of intracellular concentration would be required for *K*_d_ calculation.

KD‐FRET offers several notable strengths. First, KD‐FRET enables PPI quantification using only basic fluorescence microscopy setups, which are commonly available. The simplicity and accessibility of this technique make it broadly applicable to researchers. Moreover, KD‐FRET can be readily applied to other bacterial species that meet the following conditions: low autofluorescence in minimal media, controllable expression of fluorescent proteins with genome editing accessibility, free diffusion of proteins without cellular compartmentalization, and the ability to calculate cell volume from 2D images. Based on previous studies, we have identified *Bacillus subtilis*,^[^ ^50^ ^]^ *Caulobacter crescentus*,^[^ ^51^ ^]^ *Vibrio cholerae*,^[^ ^52^ ^]^ and *Streptococcus pneumonia* ^[^ ^53^ ^]^ as suitable candidates for KD‐FRET implementation, ensuring reliable intracellular *K*_d_ measurements in these species. In principle, the same workflow established in *E. coli*—including transformation, protein expression, and FRET measurements—can be directly applied to these bacteria. Second, KD‐FRET allows simultaneous quantification of intracellular protein concentration and FRET efficiency without cell disruption. This feature provides a direct correlation between protein concentration and interaction strength in individual living cells. Third, KD‐FRET has a *K*_d_ measurement range of ≈0.05–30 × 10^−6^ m (Note S1, Supporting Information), which encompasses the majority of PPIs occurring in *E. coli*.^[^ ^54^ ^]^ This range makes KD‐FRET highly suitable for studying a wide spectrum of biologically significant interactions in live *E. coli* cells.

The KD‐FRET method also has certain limitations. As KD‐FRET relies on FRET signals for interaction indication, the distance between the donor and acceptor FPs has to be within 7–9 nm in the protein complex. However, this requirement does not constrain the overall size of protein complex. As long as the FPs are properly positioned in a close‐proximity within the protein complex, KD‐FRET can effectively characterize interactions, even large multi‐domain proteins. Another limitation is the need for multiple control samples to perform a series of corrections. Specifically, fluorescent images of two types of cells, one expressing only the donor FP and the other expressing only the acceptor FP, are additionally required.

KD‐FRET enables unprecedented quantification of PPIs and accurate determination of FRET efficiency in living bacterial cells by successfully adapting single‐molecule FRET analysis to bacterial imaging, overcoming previous limitations of weak FRET signals.^[^ ^17^, ^20^ ^]^ This method may resolve discrepancies between PPI behaviors in vitro and in living cells, as demonstrated by our finding that certain protein pairs exhibit interactions in cells despite showing no binding in vitro. In addition, the ability to measure *K*_d_ in living *E. coli* opens new possibilities for studying how intracellular conditions or external stimuli affect PPIs. For example, KD‐FRET can quantify the effects of factors such as antibiotic exposure or metabolic shifts on the strength and stability of protein interactions in living bacterial cells, providing insights into cellular adaptability under diverse environmental conditions. In a similar example, Sukenik et al. quantified weak PPI by cell‐volume perturbation in U‐2 OS cells.^[^ ^38^ ^]^ Overall, KD‐FRET bridges the gap between in vitro studies and cellular processes, providing crucial insights for drug development, pathogen research, and biotechnology.^[^ ^55^ ^]^

## 4 Experimental Section

### Plasmids and Bacterial Strains

Plasmids and bacterial strains used in this work are listed in Tables S1 and S2 (Supporting Information). For in‐cell FRET measurement, donor and acceptor vectors were chosen with respect to plasmid incompatibility, usage of different antibiotics for selection, and inducers for individual expression of protein. As a result, pFF838 (p15A origin, chloramphenicol, anhydrotetracycline [aTc]) and pBBR6k (pBBR1 origin, kanamycin, isopropyl β‐d‐thiogalactopyranoside [IPTG]) were chosen for the donor and acceptor expression vectors, respectively. pFF838 was a gift from Timothy Lu (Addgene plasmid # 61457) and pBBR6k‐GFPuv was a gift from Brian Pfleger (Addgene plasmid # 106384). Oligonucleotides for molecular cloning were synthesized by Bionics (Table S1, Supporting Information). The DNA fragments were amplified by Primestar GXL and assembled by the HiFi DNA Assembly strategy. Fluorescent proteins were amplified from pcDNA3.1(+) eGFP, a gift from Jeremy Wilusz (Addgene plasmid # 129020) and pcDNA3‐mRFP, a gift from Doug Golenbock (Addgene plasmid # 13032). The *4CL* gene from *Arabidopsis thaliana* and *CHS* gene from *Petunia × hybrida* were used to construct the in‐cell assembly assays. Short PKS DD DNA fragments with a linker were synthesized by COSMO_GENETECH_. The heterologous *crtE* and *crtI* genes from *Xanthophyllomyces dendrorhous* and *crtB* gene from *Pantoea agglomerans* were used for lycopene production in *Saccharomyces cerevisiae* host. For SY002, SY004, and SY007 strains, the lac operon was replaced with Tsr‐FP sequence using Lambda‐RED recombination using pKD46 as previously described.^[^ ^56^ ^]^ For SY006 (*uvrA uvrB* double deletion mutant) construction, the Cas9 genome editing technique was used with the procedures that were described elsewhere.^[^ ^57^ ^]^ The sequence of the plasmid was confirmed by Sanger sequencing (Bionics). *E. coli* strain BW25993 or BL21(DE3) was transformed by the recombinant plasmid for in‐cell FRET measurement or protein expression, respectively. *S. cerevisiae* haploid strain CEN.PK2‐1C (EUROSCARF 30000A) was used as a parental strain for lycopene production.

### Protein Expression and Purification

For protein purification, the expression vector pET21c transformed BL21 (DE3) cells were grown in LB medium containing 50 µg mL^−1^ ampicillin at 37 °C with shaking. Overnight cultures were inoculated into fresh LB media and induced when the culture reached an OD_600_ of 0.6–0.7. The donor protein and eGFPd‐mRFP expression plasmids transformants were induced by addition of 1 × 10^−3^ m IPTG at 16 °C and the acceptor protein expression plasmid transformants were induced by addition of 0.5 × 10^−3^ m IPTG at 37 °C. After overnight of induction, the cells were harvested by 6000× *g* 10 min of centrifugation. His‐tagged protein expressed cells were resuspended with 20 × 10^−3^ m Tris‐HCl (pH 7.4), 400 × 10^−3^ m NaCl, 20 × 10^−3^ m imidazole, 2 × 10^−3^ m 4‐benzenesulfonyl fluoride hydrochloride (AEBSF), and 5 × 10^−3^ m 1,4‐dithiothreitol (DTT). GST‐tagged protein expressed cells were resuspended with PBS, supplemented with 0.5% N‐lauroylsarcosine, 2 × 10^−3^ m AEBSF, and 5 × 10^−3^ m DTT. The cells were disrupted by sonication and the crude lysate was centrifuged at 14000× *g* for 30 min at 4 °C. The supernatant was then loaded to Ni‐NTA agarose bead (for His‐tagged protein) or glutathione‐agarose bead (for GST‐tagged protein) and incubated for 1.5 h at 4 °C for sufficient bead binding. After several times of washing, His‐tagged proteins were eluted by elution buffer (20 × 10^−3^ m Tris‐HCl (pH 7.4), 100 × 10^−3^ m NaCl, and 500 × 10^−3^ m imidazole) and GST‐tagged proteins were cleaved from the resin by 30 U of thrombin. The proteins were concentrated by ultrafiltration using Amicon Ultra centrifugal filters.

### Cell Growth Condition for *K*_d_ Measurement in Living *E. Coli* Cells

To measure the accurate FRET signal, a single colony was grown overnight in 3 mL of LB medium at 37 °C. Overnight cultures were inoculated 1:100 to M9 medium supplemented with 0.4% glucose and 1× MEM vitamin and 1× MEM amino acids and induced with anhydrotetracycline (pFF838 vector) or/and IPTG (pBBR6k vector) until they reached an OD_600_ of ≈0.3. Following the induction, cells were harvested and transferred to fresh, pre‐warmed M9 medium supplemented with 0.4% glucose to be OD_600_ of ≈0.1 and grown without inducers for an additional 2 h to allow for the maturation of fluorescent proteins already expressed in cells.^[^ ^58^ ^]^ For the minimal expression of the SY002, SY004, and SY007 strains, cells were grown in 3 mL of M9 medium supplemented with 0.4% glucose, amino acids, and vitamins at 37 °C. The overnight M9 cultures were inoculated 1:200 into fresh M9 medium same as the overnight culture and grew 5–6 h at 37 °C until they reached an OD_600_ of ≈0.3.

### Microscopy

The cells were harvested by centrifugation (6000× *g*, 1 min) and washed with fresh M9 medium supplemented with 0.4% glucose. The cells were then placed on a 3% low melting temperature agarose gel pad (SeaPlaque GTG agarose, Lonza, #50111) and covered with a coverslip. Image acquisition was carried out at a temperature of 37 °C with the aid of a temperature controller (FCS2, Bioptechs). The samples were placed on an inverted microscope (Olympus, IX‐71) that was outfitted with a 100× oil‐immersed objective lens (Olympus) and further enhanced with a 1.6× amplification. The microscope was used to acquire phase‐contrast images and three fluorescence images (donor, acceptor, and FRET channels) at multiple positions using an EMCCD camera (iXon DU888, Andor). For the donor channel, FF02‐482/18‐25 (excitation), FF01‐525/45‐25 (emission), and Di03‐R488‐t1‐25 × 36 (dichroic mirror) were used. For the acceptor channel, FF01‐572/28‐25 (excitation), FF02‐641/75‐25 (emission), and FF593‐Di02‐25 × 36 (dichroic mirror) were used. For the FRET channel, FF02‐482/18‐25 (excitation), FF02‐641/75‐25 (emission), and FF593‐Di02‐25 × 36 (dichroic mirror) were used. Semrock provided all the necessary filters. A 488 nm diode laser (LX 150 mW, OBIS) and a 580 nm fiber laser (VFL‐P‐Series, MPB Communications Inc.) were used to excite the donor and acceptor, respectively. Metamorph software (Molecular Devices) was utilized to automate the measurements.

### Image Analysis

The images were analyzed using home‐built software (Matlab). The program extracted the total fluorescence intensity, cell size, major and minor axes of individual cells from the phase contrast by identification of the boundaries of individual cells. For in‐cell FRET measurements, image background intensity was measured by the mode of intensity per pixel in the image. The wild‐type BW25993 strain was used to subtract the autofluorescence of all samples. Donor‐ and acceptor‐only transformed cells were used to calculate the leakage and direct excitation, respectively. The γ factor was calculated by serial imaging SY004 strain before and after mRFP photobleaching. The intensity of the FRET channel of the PPI sample was corrected with the intensity of the background image, autofluorescence, leakage, direct excitation, and γ factor. After the correction, due to the small FRET signal and a series of correction steps, outlier removal was conducted regarding the threshold of the donor channel intensity. To quantify the number of intracellular proteins, minimally expressed SY002 and SY007 strains were imaged under the same imaging condition with the FRET measurements of living *E. coli* cells.

### *K*_d_ Calculation in Living Cells

For in‐cell *K*_d_ measurement, acceptor protein concentrations of single cells were binned together as equivalent, and their FRET efficiencies calculated with corrected FRET intensities and donor protein concentrations were averaged. The averaged FRET efficiency was plotted against the binned acceptor protein concentration and the fitted to the quadratic binding equation.

### In Vitro *K*_d_ Measurement

*K*_d_ measurements of the purified proteins were conducted by using Multimode plate reader (Synergy TM H1, BioTek Instruments). The donor channel (Excitation: 475 nm, Emission: 515 nm), the acceptor channel (Excitation: 580 nm, Emission: 607 nm), and the FRET channel (Excitation: 475 nm, Emission: 607 nm) were used. The concentration of the donor protein was fixed to 0 and 1 × 10^−6^ m and that of the acceptor protein was increased 0 to 20 × 10^−6^ m. In the case of in vitro *K*_d_ calculation, binning of the acceptor protein concentration was unnecessary and the donor protein concentration was fixed to 1 × 10^−6^ m.

### In Vitro FRET Efficiency Measurement of Purified Positive Control

The FRET efficiency can be calculated considering the extinction coefficients of donor and acceptor, as follows:^[^ ^20^, ^34^, ^59^ ^]^

(4) $$E=\frac{F_{DA}^{FRET}}{F_{AA}^{FRET}}*\frac{\varepsilon_{A_{ex}}^{A}}{\varepsilon_{D_{ex}}^{D}}$$

where $F_{DA}^{FRET}$ and $F_{AA}^{FRET}$ are the FRET channel intensity and the acceptor channel intensity of sample, respectively. $\varepsilon_{A_{ex}}^{A}$ is the extinction coefficient of the acceptor protein at acceptor excitation wavelength, and $\varepsilon_{D_{ex}}^{D}$ is the extinction coefficient of the donor protein at donor excitation wavelength. Leakage and direct excitation were corrected from the FRET channel intensities using purified eGFPd and mRFP. The intensities of the FRET channel and the acceptor channel of purified samples were measured using a fluorescence spectrophotometer (Cary Eclipse, Agilent). To identify the molar extinction coefficients of eGFPd and mRFP at the wavelengths used in this work, the concentrations of the proteins were determined by a BCA assay, and the absorbance was measured using a UV–vis spectrophotometer (Libra S70, Biochrom).

### Isothermal Titration Calorimetry

The ITC sample cell was filled with 400 µL of 80 × 10^−6^ m eGFPd‐^C^DD_MlnB_, prepared in a buffer containing 20 × 10^−3^ m Tris‐HCl (pH 7.9), 250 × 10^−3^ m NaCl, 1 × 10^−3^ m EDTA, 10% (v/v) glycerol, and 1 × 10^−3^ m TCEP. Using a micro‐syringe, 2 µL of 1.6 × 10^−3^ m ^N^DD_MlnC_‐mRFP was injected into the sample cell at 400 s intervals with gentle stirring. A total 20 injections were titrated for each measurement. All experiments were performed at 37 °C using the Affinity ITC instruments (TA Instruments, New Castle, DE). The experimental data were fitted assuming a single binding site model.

### Metabolic Assays in *E. Coli* and *S. Cerevisiae*

Plasmids and strains for in‐cell assay of 4CL‐CHS assembly mediated by DD pairs are listed in Tables S2 and S3 (Supporting Information). For comparison, constructs of 4CL‐CHS combinations were also prepared for cases without DD and with only acceptor DD or donor DD. The relevant plasmids were transformed into *E. coli* BW25993 for naringenin production. The recombinant *E. coli* strains were grown in LB medium supplemented with 50 µg mL^−1^ kanamycin at 37 °C until an OD600 of 0.8. The cells were re‐cultured in 50 mL of YM9 medium (6.8 g of Na_2_HPO_4_, 3 g of KH_2_PO_4_, 0.5 g of NaCl, 1 g of NH_4_Cl, 10 g of yeast extract, 30 mL of glycerol, 42 g of MOPS (pH 7.0) per liter) supplemented with 50 µg mL^−1^ kanamycin at 37 °C. After an OD600 of 0.8 had been reached, the expression was induced with 0.1 × 10^−3^ m IPTG, and 1 × 10^−3^ m *p*‐coumaric acid was added as the initial substrate for naringin biosynthesis, followed by incubation at 30 °C for 40 h. To analyze naringenin production, the culture broth was extracted with twice the volume of ethyl acetate and evaporated to dryness, and the extracts were dissolved in methanol. Samples were analyzed by ultra‐performance liquid chromatography (UPLC)–quadrupole time‐of‐flight high‐resolution mass spectrometry (qTOF‐HR‐MS), as described in a previous work.^[^ ^60^ ^]^ Independent experiments were performed three or more times.

Plasmids and strains for assembly of lycopene pathway enzymes are listed in Tables S2 and S3 (Supporting Information). For lycopene production, *S. cerevisiae* strains were cultivated in synthetic complete (SC) medium containing 20 g L^−1^ glucose, 6.7 g L^−1^ yeast nitrogen base without amino acids, and a suitable concentration of amino acid dropout mixture for plasmid selection. Seed cultures were initiated by inoculating yeast cells harboring plasmids into 5 mL of selective SC medium in a 50‐mL flask, followed by incubation at 30 °C for 24 h with shaking at 170 rpm. Subsequently, 10 mL of selective SC medium in a 100‐mL flask was inoculated with the seed culture at an initial OD_600_ of 0.2 and incubated under the same conditions for 120 h. Lycopene extraction was performed using a customized hot‐HCl method. Cultured yeast cells were resuspended in 1 N HCl and lysed by alternating boiling and cooling cycles. Specifically, the cells were subjected to 7 min of boiling, followed by 3 min on ice, another 7 min of boiling, and a final 5 min on ice. After cooling, the lysed cells were washed with triple‐distilled water (TDW) and collected by centrifugation for 3 min. Lycopene was extracted by adding an appropriate volume of HPLC‐grade acetone to the cell pellet, followed by vortexing for 5 min. Cell debris was removed by centrifugation for 5 min, and the resulting supernatant was used for lycopene quantification. Lycopene quantification was performed using an UltiMate 3000 HPLC system (Thermo Fisher Scientific) equipped with an Agilent Eclipse XDB‐C18 column (250 mm × 4.6 mm, 5 µm, Agilent, Santa Clara, CA). Filtered samples were analyzed with a mobile phase composed of dichloromethane:acetonitrile:methanol in a ratio of 16:42:42, at a flow rate of 1.2 mL min^−1^. Lycopene detection was achieved using a UV–visible detector set to a wavelength of 471 nm. The column temperature was maintained at 30 °C throughout the analysis.

### Statistical Analysis

All experiments were performed in triplicate, and the results are presented as mean ± standard deviation (SD). The *K*_d_ values were determined by fitting the FRET efficiency versus acceptor concentration data to a quadratic binding equation using nonlinear regression analysis. For plotting the KD‐FRET data, 95% confidence intervals were calculated and displayed as shaded regions to represent the variability within each bin. For naringenin production experiments, statistical significance was determined using one‐way ANOVA followed by Tukey's post‐hoc test. *P*‐values less than 0.05 were considered statistically significant.

All statistical analyses and graph plotting were performed using Origin 2023b (OriginLab, Northampton, MA) or Microsoft Excel 2016 software.

## Conflict of Interest

The authors declare no conflict of interest.

## Author Contributions

S.Y. and E.K. contributed equally to this work. S.Y., E.K., Y.J.Y., and N.K.L. conceived and designed the study. S.Y., S.Y., and E.K. performed FRET measurements and analyzed the data. E.K., G.K., and J.‐S.H. conducted artificial metabolon experiments and data analysis. J.S.J. and H.H.L. carried out the ITC experiments. D.‐W.B., S.‐Y.S., B.‐G.J., and S.‐S.C. contributed to data analysis. S.Y., E.K., J.‐S.H., Y.J.Y., and N.K.L. wrote the manuscript, which was reviewed and approved by all authors for the final version. Y.J.Y. and N.K.L. supervised the project.

## Supporting information



Supporting Information

## Data Availability

The data that support the findings of this study are available from the corresponding author upon reasonable request.

## References

[1] a) K. M. Poluri , K. Gulati , D. K. Tripathi , N. Nagar , Protein‐Protein Interactions, Springer Nature, Berlin 2023;

[2] a) G. Papadakos , A. Sharma , L. E. Lancaster , R. Bowen , R. Kaminska , A. P. Leech , D. Walker , C. Redfield , C. Kleanthous , J. Am. Chem. Soc. 2015, 137, 5252; 25856265 10.1021/ja512607r

[3] M. R. Arkin , A. Whitty , Curr. Opin. Chem. Biol. 2009, 13, 284. 19553156 10.1016/j.cbpa.2009.05.125

[4] a) N. K. Aghera , J. Prabha , H. Tandon , G. Chattopadhyay , S. Vishwanath , N. Srinivasan , R. Varadarajan , Structure 2020, 28, 562; 32294467 10.1016/j.str.2020.03.006

[5] S. L. Speer , W. Zheng , X. Jiang , I. T. Chu , A. J. Guseman , M. Liu , G. J. Pielak , C. Li , Proc. Natl. Acad. Sci. USA 2021, 118, e2019918118. 33836588 10.1073/pnas.2019918118 PMC7980425

[6] a) I. Piazza , K. Kochanowski , V. Cappelletti , T. Fuhrer , E. Noor , U. Sauer , P. Picotti , Cell 2018, 172, 358; 29307493 10.1016/j.cell.2017.12.006

[7] T. C. Jarvis , D. M. Ring , S. S. Daube , P. H. Vonhippel , J. Biol. Chem. 1990, 265, 15160. 2168402

[8] a) S. Xing , N. Wallmeroth , K. W. Berendzen , C. Grefen , Plant Physiol. 2016, 171, 727; 27208310 10.1104/pp.16.00470 PMC4902627

[9] a) A. M. Edwards , B. Kus , R. Jansen , D. Greenbaum , J. Greenblatt , M. Gerstein , Trends Genet. 2002, 18, 529; 12350343 10.1016/s0168-9525(02)02763-4

[10] J. Mehla , J. H. Caufield , N. Sakhawalkar , P. Uetz , in Methods in Enzymology, Vol. 586 (Ed: A. K. Shukla ), Academic Press, San Diego, CA 2017, pp. 333–358. 28137570 10.1016/bs.mie.2016.10.020 PMC5737774

[11] Y. Hu , K. Cheng , L. He , X. Zhang , B. Jiang , L. Jiang , C. Li , G. Wang , Y. Yang , M. Liu , Anal. Chem. 2021, 93, 1866. 33439619 10.1021/acs.analchem.0c03830

[12] a) E. J. Osterlund , N. Hirmiz , J. M. Pemberton , A. Nougarede , Q. Liu , B. Leber , Q. Fang , D. W. Andrews , Sci. Adv. 2022, 8, eabm7375; 35442739 10.1126/sciadv.abm7375 PMC9020777

[13] D. C. Youvan , W. J. Coleman , C. M. Silva , E. J. Bylina , M. R. Dilworth , M. M. Yang , Biotechnology 1997, 3, 1.

[14] G. W. Gordon , G. Berry , X. H. Liang , B. Levine , B. Herman , Biophys. J. 1998, 74, 2702. 9591694 10.1016/S0006-3495(98)77976-7 PMC1299610

[15] Z. Xia , Y. Liu , Biophys. J. 2001, 81, 2395. 11566809 10.1016/S0006-3495(01)75886-9 PMC1301710

[16] A. Coullomb , C. M. Bidan , C. Qian , F. Wehnekamp , C. Oddou , C. Albiges‐Rizo , D. C. Lamb , A. Dupont , Sci. Rep. 2020, 10, 6504. 32300110 10.1038/s41598-020-62924-w PMC7162988

[17] X. You , A. W. Nguyen , A. Jabaiah , M. A. Sheff , K. S. Thorn , P. S. Daugherty , Proc. Natl. Acad. Sci. USA 2006, 103, 18458. 17130455 10.1073/pnas.0605422103 PMC1693684

[18] V. Sourjik , H. C. Berg , Proc. Natl. Acad. Sci. USA 2002, 99, 12669. 12232047 10.1073/pnas.192463199 PMC130518

[19] T. Lin , B. L. Scott , A. D. Hoppe , S. Chakravarty , Prot. Sci. 2018, 27, 1850. 10.1002/pro.3482 PMC6199148 30052312

[20] N. K. Lee , A. N. Kapanidis , Y. Wang , X. Michalet , J. Mukhopadhyay , R. H. Ebright , S. Weiss , Biophys. J. 2005, 88, 2939. 15653725 10.1529/biophysj.104.054114 PMC1282518

[21] G. Agam , C. Gebhardt , M. Popara , R. Machtel , J. Folz , B. Ambrose , N. Chamachi , S. Y. Chung , T. D. Craggs , M. de Boer , D. Grohmann , T. Ha , A. Hartmann , J. Hendrix , V. Hirschfeld , C. G. Hubner , T. Hugel , D. Kammerer , H. S. Kang , et al., Nat. Methods 2023, 20, 523. 36973549 10.1038/s41592-023-01807-0 PMC10089922

[22] R. P. Novick , Microbiol. Rev. 1987, 51, 381. 3325793 10.1128/mr.51.4.381-395.1987 PMC373122

[23] G. N. van der Krogt , J. Ogink , B. Ponsioen , K. Jalink , PLoS One 2008, 3, e1916. 18382687 10.1371/journal.pone.0001916 PMC2271053

[24] a) C. Wu , M. Mori , M. Abele , A. Banaei‐Esfahani , Z. Zhang , H. Okano , R. Aebersold , C. Ludwig , T. Hwa , Nat. Microbiol. 2023, 8, 347; 36737588 10.1038/s41564-022-01310-w PMC9994330

[25] a) J. B. Son , S. Kim , S. Yang , Y. Ahn , N. K. Lee , J. Phys. Chem. B 2024, 128, 6730; 38968413 10.1021/acs.jpcb.4c01816 PMC11264270

[26] C. Berney , G. Danuser , Biophys. J. 2003, 84, 3992. 12770904 10.1016/S0006-3495(03)75126-1 PMC1302980

[27] a) T. Ha , J. Fei , S. Schmid , N. K. Lee , R. L. Gonzalez , S. Paul , S. Yeou , Nat. Rev. Methods Primers 2024, 4, 21;

[28] a) H. Chen , H. L. Puhl III, S. V. Koushik , S. S. Vogel , S. R. Ikeda , Biophys. J. 2006, 91, L39; 16815904 10.1529/biophysj.106.088773 PMC1544280

[29] a) T. Ha , A. Y. Ting , J. Liang , W. B. Caldwell , A. A. Deniz , D. S. Chemla , P. G. Schultz , S. Weiss , Proc. Natl. Acad. Sci. USA 1999, 96, 893; 9927664 10.1073/pnas.96.3.893 PMC15321

[30] B. Epe , K. G. Steinhauser , P. Woolley , Proc. Natl. Acad. Sci. USA 1983, 80, 2579. 16593305 10.1073/pnas.80.9.2579 PMC393869

[31] S. Yang , S. Kim , Y. R. Lim , C. Kim , H. J. An , J. H. Kim , J. Sung , N. K. Lee , Nat. Commun. 2014, 5, 4761. 25175593 10.1038/ncomms5761

[32] I. Jarmoskaite , I. AlSadhan , P. P. Vaidyanathan , D. Herschlag , Elife 2020, 9, e57264. 32758356 10.7554/eLife.57264 PMC7452723

[33] A. Plochowietz , R. Crawford , A. N. Kapanidis , Phys. Chem. Chem. Phys. 2014, 16, 12688. 24837080 10.1039/c4cp00995a

[34] R. M. Clegg , Methods Enzymol. 1992, 211, 353. 1406315 10.1016/0076-6879(92)11020-j

[35] S. Y. Son , D. W. Bae , E. Kim , B. G. Jeong , M. Y. Kim , S. Y. Youn , S. Yi , G. Kim , J. S. Hahn , N. K. Lee , Y. J. Yoon , S. S. Cha , Structure 2024, 32, 1477. 38908377 10.1016/j.str.2024.05.017

[36] J. L. Meinke , A. J. Simon , D. T. Wagner , B. R. Morrow , S. You , A. D. Ellington , A. T. Keatinge‐Clay , ACS Synth. Biol. 2019, 8, 2017. 31469555 10.1021/acssynbio.9b00047 PMC7102495

[37] a) S. B. Zimmerman , S. O. Trach , J. Mol. Biol. 1991, 222, 599; 1748995 10.1016/0022-2836(91)90499-v

[38] S. Sukenik , P. Ren , M. Gruebele , Proc. Natl. Acad. Sci. USA 2017, 114, 6776. 28607089 10.1073/pnas.1700818114 PMC5495242

[39] D. Pakotiprapha , M. Samuels , K. Shen , J. H. Hu , D. Jeruzalmi , Nat. Struct. Mol. Biol. 2012, 19, 291. 22307053 10.1038/nsmb.2240

[40] a) E. S. Day , S. M. Cote , A. Whitty , Biochemistry 2012, 51, 9124; 23088250 10.1021/bi301039t PMC3567247

[41] a) D. Roberts , R. Keeling , M. Tracka , C. F. van der Walle , S. Uddin , J. Warwicker , R. Curtis , Mol. Pharm. 2015, 12, 179; 25389571 10.1021/mp500533c

[42] a) J. Zeng , D. T. Wagner , Z. Zhang , L. Moretto , J. D. Addison , A. T. Keatinge‐Clay , ACS Chem. Biol. 2016, 11, 2466; 27362945 10.1021/acschembio.6b00345 PMC5460766

[43] a) J. Behrendorff , G. Borras‐Gas , M. Pribil , Trends Biotechnol. 2020, 38, 432; 31718802 10.1016/j.tibtech.2019.10.009

[44] a) Y. Zhang , S. Z. Li , J. Li , X. Pan , R. E. Cahoon , J. G. Jaworski , X. Wang , J. M. Jez , F. Chen , O. Yu , J. Am. Chem. Soc. 2006, 128, 13030; 17017764 10.1021/ja0622094

[45] a) Y. Zang , J. Zha , X. Wu , Z. Zheng , J. Ouyang , M. A. G. Koffas , J. Agric. Food Chem. 2019, 67, 13430; 30919618 10.1021/acs.jafc.9b00413

[46] Z. Serber , R. Ledwidge , S. M. Miller , V. Dotsch , J. Am. Chem. Soc. 2001, 123, 8895. 11552796 10.1021/ja0112846

[47] a) B. Wallace , P. J. Atzberger , PLoS One 2017, 12, e0177122; 28542211 10.1371/journal.pone.0177122 PMC5438121

[48] a) G. Karimova , J. Pidoux , A. Ullmann , D. Ladant , Proc. Natl. Acad. Sci. USA 1998, 95, 5752; 9576956 10.1073/pnas.95.10.5752 PMC20451

[49] a) D. S. Burz , K. Dutta , D. Cowburn , A. Shekhtman , Nat. Methods 2006, 3, 91; 16432517 10.1038/nmeth851 PMC4447212

[50] a) R. McQuillen , A. J. Perez , X. Yang , C. H. Bohrer , E. L. Smith , S. Chareyre , H.‐C. T. Tsui , K. E. Bruce , Y. M. Hla , J. W. McCausland , M. E. Winkler , E. D. Goley , K. S. Ramamurthi , J. Xiao , Nat. Commun. 2024, 15, 10746; 39737933 10.1038/s41467-024-54974-9 PMC11685620

[51] a) A. Persat , H. A. Stone , Z. Gitai , Nat. Commun. 2014, 5, 3824; 24806788 10.1038/ncomms4824 PMC4104588

[52] S. Vaidya , D. Saha , D. K. H. Rode , G. Torrens , M. F. Hansen , P. K. Singh , E. Jelli , K. Nosho , H. Jeckel , S. Göttig , F. Cava , K. Drescher , Nat. Microbiol. 2025, 10, 144. 39753671 10.1038/s41564-024-01886-5 PMC11726461

[53] a) A. J. Perez , Y. Cesbron , S. L. Shaw , J. Bazan Villicana , H. T. Tsui , M. J. Boersma , Z. A. Ye , Y. Tovpeko , C. Dekker , S. Holden , M. E. Winkler , Proc. Natl. Acad. Sci. USA 2019, 116, 3211; 30718427 10.1073/pnas.1816018116 PMC6386697

[54] J. R. Perkins , I. Diboun , B. H. Dessailly , J. G. Lees , C. Orengo , Structure 2010, 18, 1233. 20947012 10.1016/j.str.2010.08.007

[55] a) D. Schultz , M. Stevanovic , L. S. Tsimring , Biophys. J. 2022, 121, 4137; 36168291 10.1016/j.bpj.2022.09.028 PMC9675034

[56] K. A. Datsenko , B. L. Wanner , Proc. Natl. Acad. Sci. USA 2000, 97, 6640. 10829079 10.1073/pnas.120163297 PMC18686

[57] J. Y. Seok , Y. H. Han , J. S. Yang , J. Yang , H. G. Lim , S. G. Kim , S. W. Seo , G. Y. Jung , Cell Rep. 2021, 36, 109589. 34433019 10.1016/j.celrep.2021.109589

[58] a) D. C. Prasher , V. K. Eckenrode , W. W. Ward , F. G. Prendergast , M. J. Cormier , Gene 1992, 111, 229; 1347277 10.1016/0378-1119(92)90691-h

[59] V. Mekler , E. Kortkhonjia , J. Mukhopadhyay , J. Knight , A. Revyakin , A. N. Kapanidis , W. Niu , Y. W. Ebright , R. Levy , R. H. Ebright , Cell 2002, 108, 599. 11893332 10.1016/s0092-8674(02)00667-0

[60] M. S. Xiao , J. E. Wilusz , Nucleic Acids Res. 2019, 47, 8755. 31269210 10.1093/nar/gkz576 PMC6895279

