# Supplementary material for: Direct Quantification of Protein–Protein Interactions in Living Bacterial Cells
Source: Adv Sci (Weinh). 2025 Mar 24;12(19):2414777. doi: 10.1002/advs.202414777 (PMC12097012; doi:10.1002/advs.202414777)
Supplement: Supplementary file 1 — Supporting Information [file ADVS-12-2414777-s001.docx]

Direct Quantification of Protein-Protein Interactions in Living Bacterial Cells

Soojung Yi^+^, Eunji Kim^+^, Sora Yang, Gyeongmin Kim, Da-Woon Bae, Se-Young Son, Bo-Gyeong Jeong, Jeong Seok Ji, Hyung Ho Lee, Ji-Sook Hahn, Sun-Shin Cha, Yeo Joon Yoon,* Nam Ki Lee*

[*] S. Yi,^+^ Dr. S. Yang, J. S. Ji, Prof. Dr. H. H. Lee, Prof. Dr. N. K. Lee
Department of Chemistry, Seoul National University
1 Gwanak-ro, Gwanak-gu, 08826 Seoul (Republic of Korea)
E-mail: namkilee@snu.ac.kr

Dr. E. Kim,^+^ Prof. Dr. Y. J. Yoon
Natural Products Research Institute, College of Pharmacy, Seoul National University
1 Gwanak-ro, Gwanak-gu, Seoul 08826 (Republic of Korea)
E-mail: yeojoonyoon@snu.ac.kr
G. Kim, Prof. Dr. J.-S. Hahn
School of Chemical and Biological Engineering, Institute of Chemical Processes, Seoul National University
1 Gwanak-ro, Gwanak-gu, 08826 Seoul (Republic of Korea)

Dr. D.-W. Bae, S.-Y. Son, Dr. B.-G. Jeong, Prof. Dr. S.-S. Cha

Department of Chemistry and Nanoscience, Ewha Womans University
52 Ewhayeodae-gil, Seodaemun-gu, Seoul 03760 (Republic of Korea)

[^+^] These authors contributed equally to this work.

**Supplementary Note**

**Quantifying intracellular protein concentration**

Nondisruptive measurement of the intracellular protein concentration is important for accurate K_d_ determination. Taking advantage of the fusion of FPs with the protein of interest, the number of proteins within the cell can be easily measured by assessing the intensity of a single FP. When FPs are expressed alone, their rapid diffusion throughout the cell results in a blurry fluorescence signal. However, when FPs are fused with membrane proteins and expressed under repressed conditions, the FP-fused membrane proteins are inserted into the membrane and detected as a diffraction-limited fluorescent spot.^[1]^ By conducting consecutive imaging and subsequently photobleaching the spot in a stepwise manner, the pixel intensity of a single FP can be obtained (Figure S4A). In this study, we fused eGFPd or mRFP to the C terminus of Tsr, a membrane protein transported to the inner membrane. The fused gene was inserted in place of the native *lacZ* gene, which was controlled by the lac promoter. Under our imaging conditions, the single eGFPd and mRFP intensities per pixel were measured to be 95 ± 40.2 and 32 ± 9, respectively (Figure S4B). The number of FP-fused proteins can be calculated using the following formula:

$$N_{FP fused protein}= \frac{Intracellular I_{DD} or I_{AA}}{Single FP intensity}$$

, where $I_{DD}$ is the donor channel intensity and $I_{AA}$ is the acceptor channel intensity of a single cell.^[2]^ Detailed concept of image analysis is described in Experimental section.

It is necessary to determine the volume of each cell to obtain K_d_ in units of concentration. This can be achieved by converting the 2D area obtained via phase contrast images into the 3D volume. Under our experimental conditions, the width of a single *E. coli* cell exhibited a relatively constant cell width.^[3]^ Therefore, the area of a cell can be converted into volume using the following equation (Figure S5):

$$V=r*\pi\left( \frac{4}{3}*r^{2}+{\frac{A-\pi*r}{2}}^{2} \right)$$

where V is the single-cell volume, r is half the cell width, and A is the cell area. In this work, r is measured to be 494 ± 12 nm (Figure S5). The concentration of intracellular proteins was calculated by dividing the number of FP-fused proteins by the cell volume.

**The range of K_d_ values measurable by KD-FRET**

The range of K_d_ values measurable by KD-FRET approach is determined by two factors: the sensitivity of fluorescence detection and the achievable protein expression levels (protein concentrations) without inducing the formation of inclusion bodies (protein aggregates at high concentrations) within living *E. coli* cells.

First, the lower limit of K_d_. A single copy of a fluorescent protein (FP) can typically be detected in a living *E. coli* cell.^[1]^ Assuming a FRET efficiency of 0.2, the FRET signals from five copies of acceptor FP would be equivalent to the fluorescence intensity of a copy of acceptor FP. To measure K_d_ quantitatively, protein concentrations have to vary from 0.1*K_d_ to 10*K_d_. This means that the K_d_ value corresponding to approximately 50 copies of the acceptor FP may represent the lower limit. Given that the concentration of a single protein molecule in a bacterial cell volume (~1 fL) is approximately 1 nM, these factors collectively indicate that the lower limit for K_d_ measurement using KD-FRET is approximately 50 nM (0.05 μM). It is worth noting that this lower limit is not unique to KD-FRET but represents a fundamental limitation of any FRET-based approach.

Second, the upper limit of K_d_ measurement is determined by the onset of inclusion body formation caused by protein overexpression in *E. coli*. Previous studies have shown that when the total concentration of a protein of interest exceeds approximately 2% of the total cellular protein content, inclusion bodies begin to form.^[4]^ Typically, inclusion bodies are generated at protein concentrations around 200-300 µM. Considering that protein concentrations have to be varied from 0.1*K_d_ to 10*K_d_ for quantitative measurement, this sets the upper limit of K_d_ detection at approximately 20-30 µM. However, if a protein of interest remains stable at higher concentrations without forming inclusion bodies, K_d_ values larger than 30 µM can be measured using KD-FRET.

**Supplementary Figures**


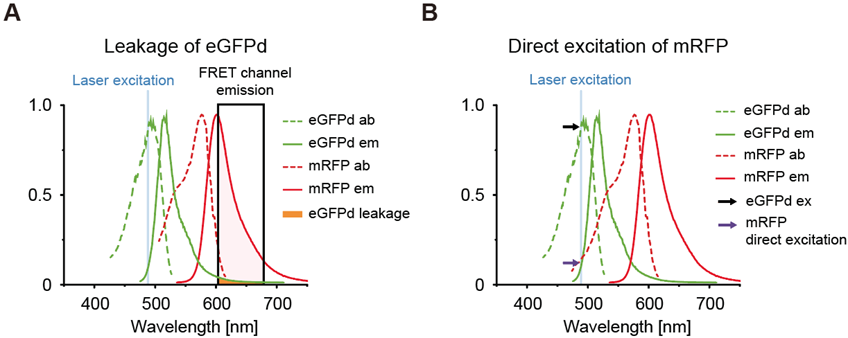


**Figure S1. Illustration of spectral crosstalks in FRET measurements.** The absorption (ab, dashed line) and emission (em, solid line) spectra of eGFPd (green) and mRFP (red) are shown. The blue line marks the wavelength of laser excitation (488 nm).

(A) Leakage of eGFPd emission into the FRET channel. The orange area indicates eGFPd emission in the FRET emission window, which causes leakage.

(B) Direct excitation of mRFP by the donor excitation laser. The purple arrow indicates that mRFP absorbs the laser at 488 nm. Thus, mRFP can be excited by the donor excitation laser (488 nm), which contributes to the FRET channel signal despite the laser being primarily optimized for eGFPd excitation. The black arrow indicates the excitation of eGFPd by the 488 nm laser.

**
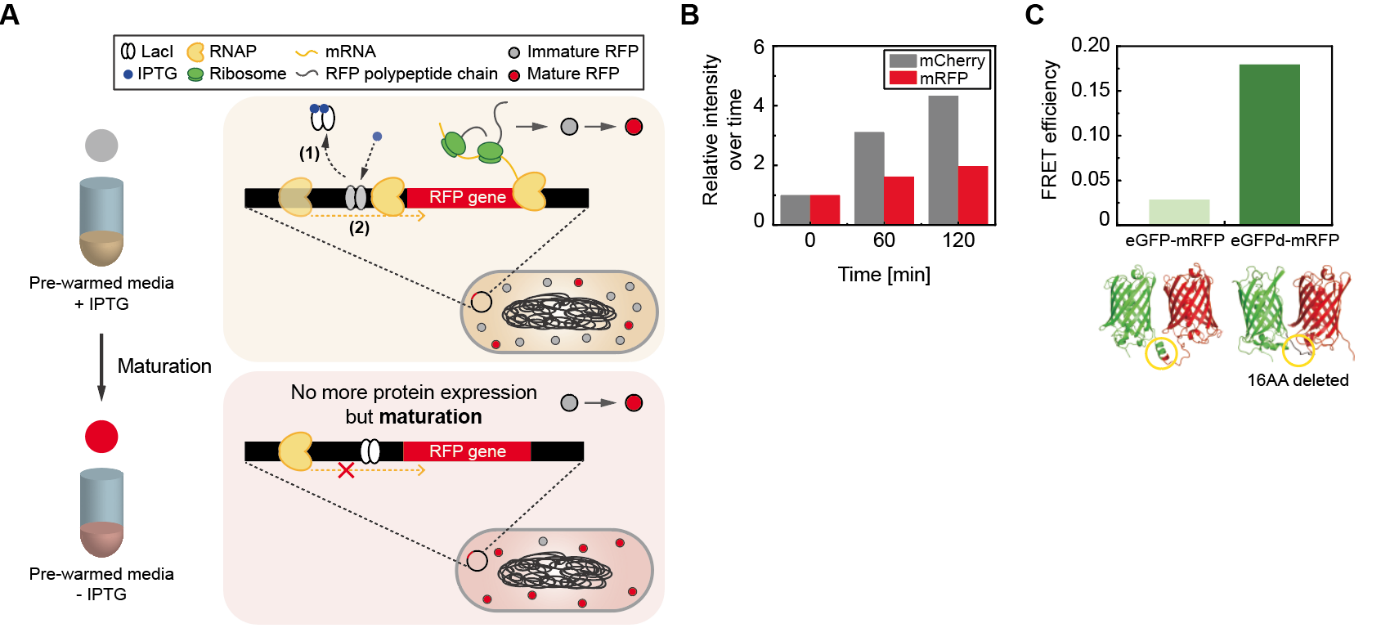
**

**Figure S2. The expression and maturation of FPs, and the selection of FPs in this study.**

(A) Schematic of the experimental setup for monitoring protein maturation. After initial induction with IPTG, protein expression is halted by removing IPTG from the medium, allowing only protein maturation to continue.

(B) Relative fluorescence intensity over time for mCherry and mRFP, showing the maturation kinetics of each fluorescent protein. mCherry exhibits slower maturation compared to mRFP.

(C) Comparison of FRET efficiency between eGFP-mRFP and eGFPd-mRFP constructs. Deletion of 16 amino acids from the C-terminus of eGFP (eGFPd) increases FRET efficiency.


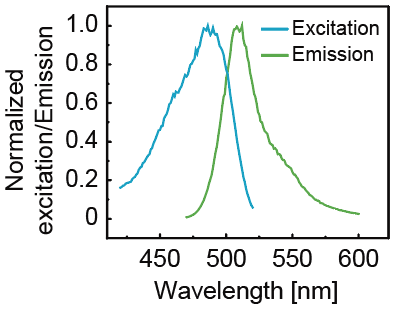


Figure S3. Normalized excitation and emission spectra of eGFPd.

Normalized excitation and emission spectra of eGFPd, demonstrating the optical properties of the donor fluorescent protein used in this work. The excitation peak is at approximately 488 nm, and the emission peak is at approximately 512 nm.


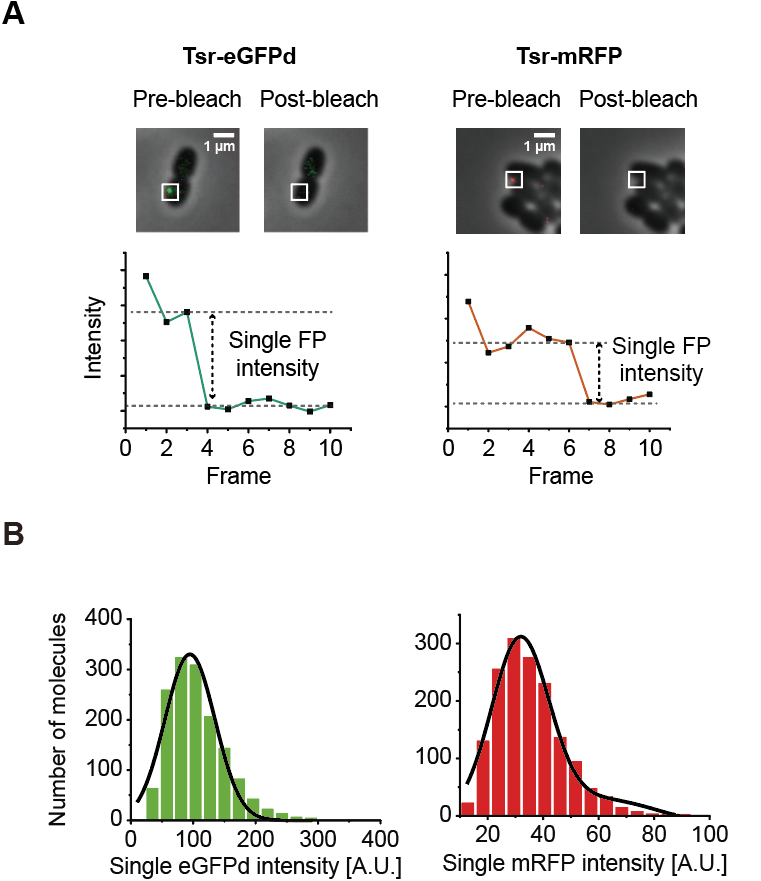


Figure S4. Tsr-FP system to quantify the number of intracellular proteins.

(A) Schematic representation of the Tsr-eGFPd and Tsr-mRFP systems used for quantifying intracellular protein concentrations through photobleaching. Fluorescent spots corresponding to individual Tsr-eGFPd or Tsr-mRFP molecules were monitored before and after photobleaching. (Scale bar = 1 µm)

(B) Histograms showing the distribution of single eGFPd and mRFP intensities, as calculated from the stepwise photobleaching measurements (n=1511 for eGFPd, n=1175 for mRFP). The single FP intensities were used for quantifying the number of proteins in each cell.


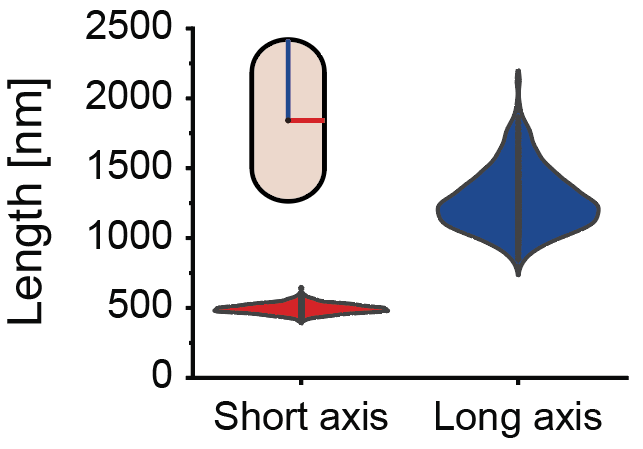


Figure S5. Calculation of cellular dimensions for volume estimation.

Violin plot shows the distribution of lengths measured for the short and long axes of each *E. coli* cell from the phase contrast images (n=359). The lengths were used for the volume calculations of each cell.


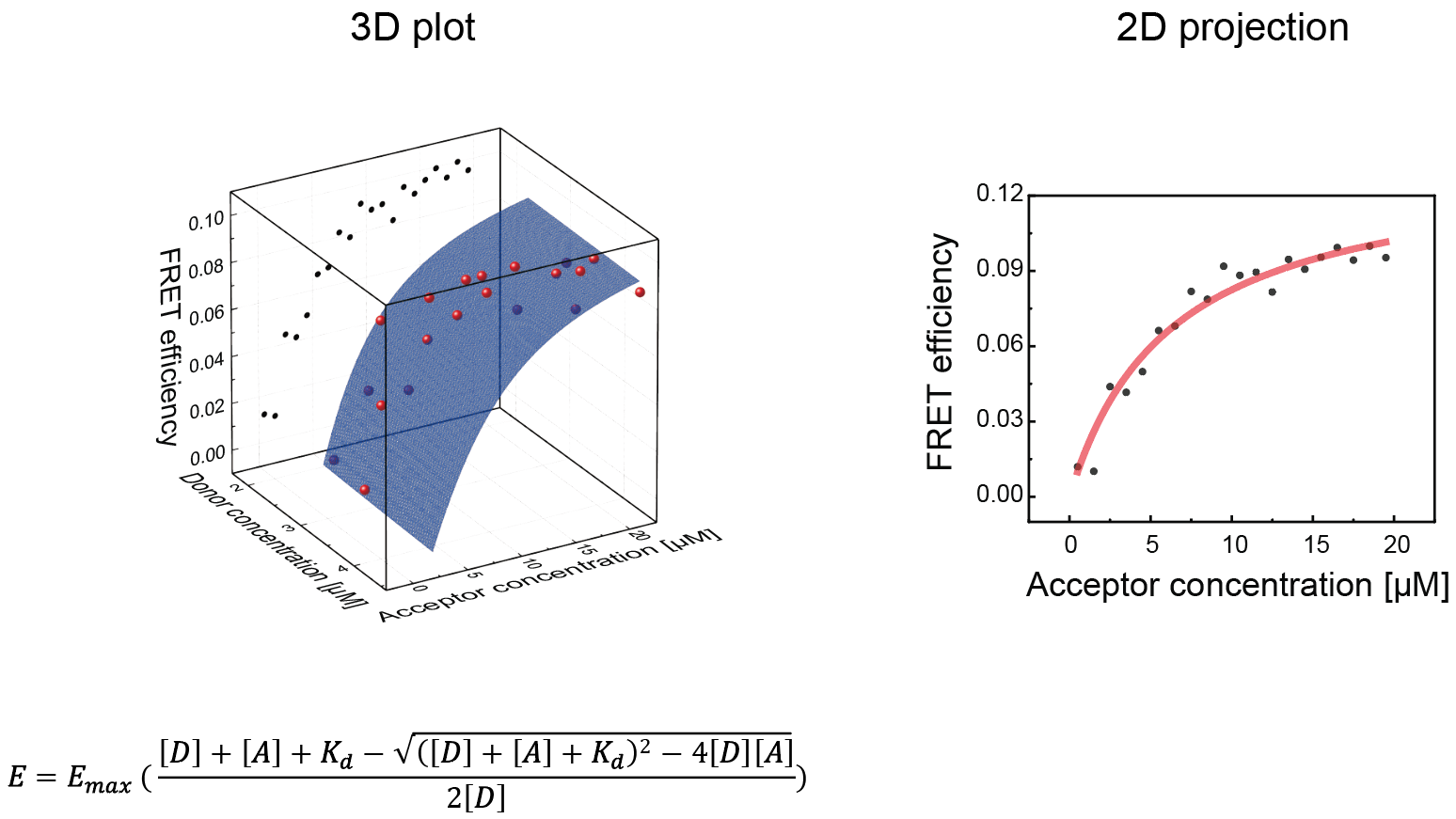


Figure S6. Determination of dissociation constant from the FRET efficiency.

(Left) 3D plot showing FRET efficiency as a function of donor and acceptor concentrations. (Right) 2D projection of the FRET efficiency versus acceptor concentration, fitted with a quadratic binding equation to determine the K_d_ values.


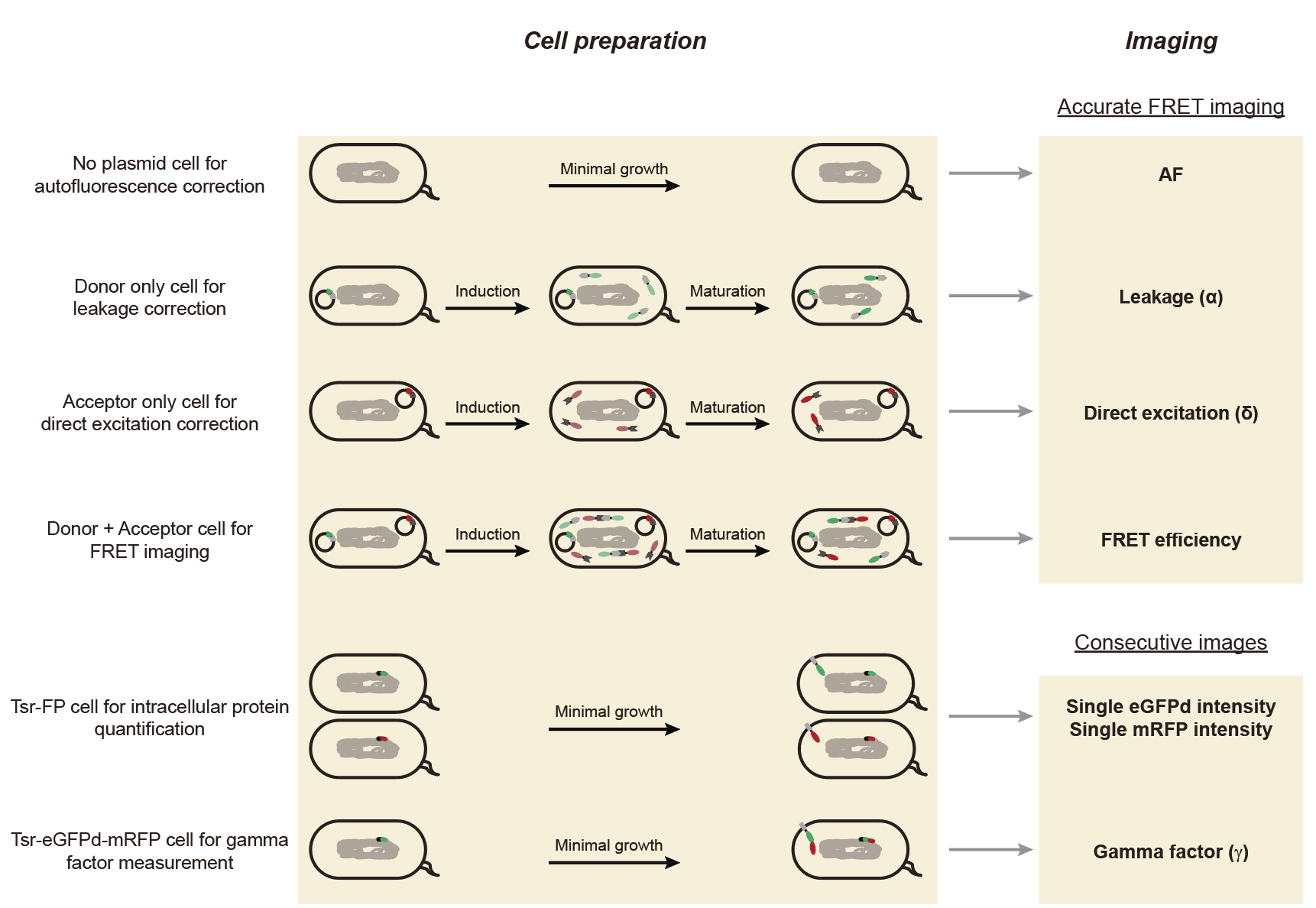


Figure S7. Detailed workflow for accurate FRET measurement and data correction.

Step-by-step workflow for preparing *E. coli* cells and performing accurate FRET measurement. The workflow includes cell preparation for autofluorescence correction, leakage and direct excitation correction and γ factor measurement, followed by imaging protocols for each step. The figure summarizes the systematic approach used to ensure reliable and accurate FRET measurements in living cells.


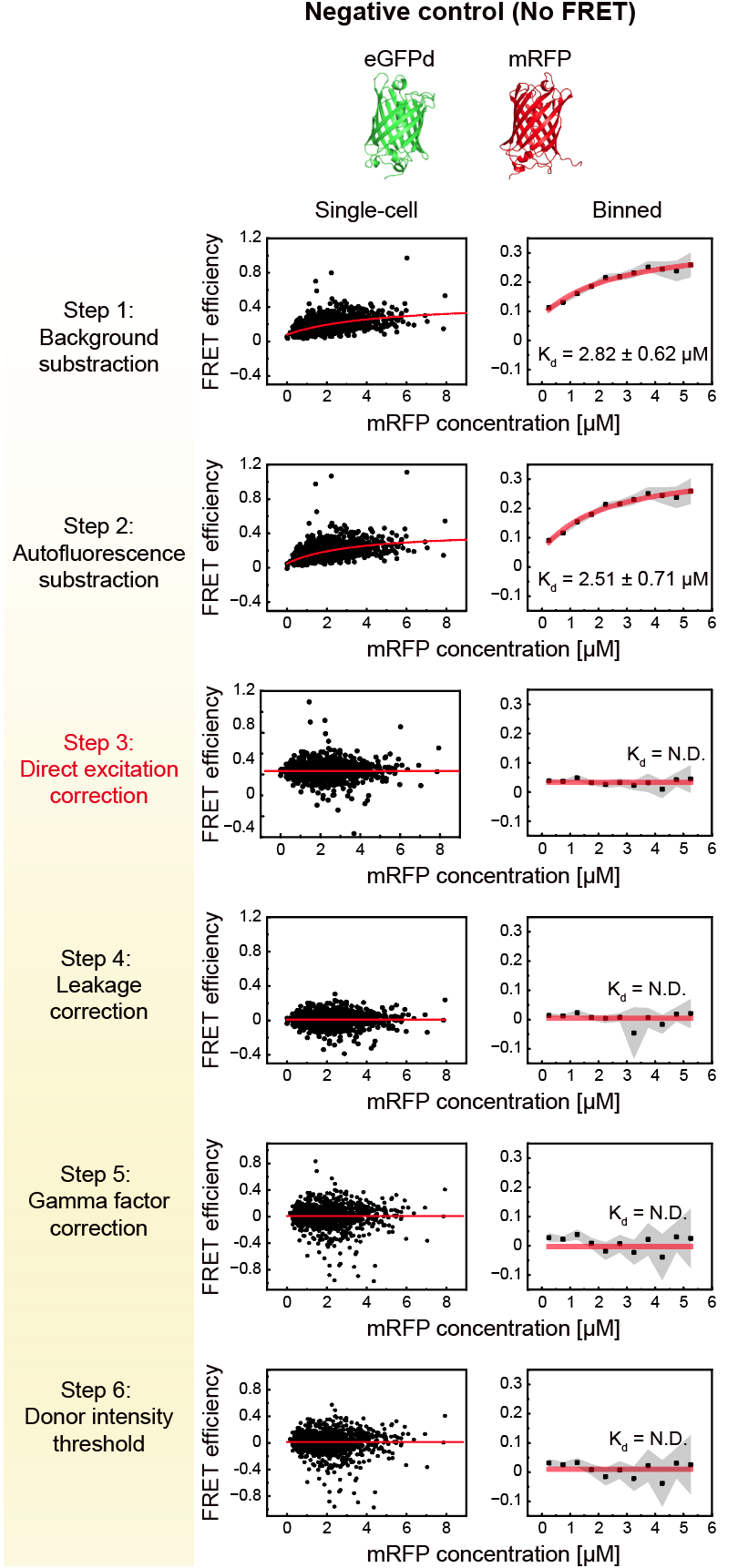


Figure S8. Effect of applying direct excitation correction before leakage correction.

FRET efficiency and K_d_ measurements at each correction step for negative and positive control pairs, with the direct excitation correction applied before the leakage correction (step 3 and 4). The plots illustrate the role of direct excitation correction in minimizing false positive K_d_ values, as seen in the negative control. Applying direct excitation correction at an earlier stage eliminated the detection of false interactions. K_d_ values are reported as mean ± standard deviation (SD) of triplicates. The gray-shaded regions represent the 95% confidence intervals for the binned mean FRET efficiency. N.D.: Not Determined.


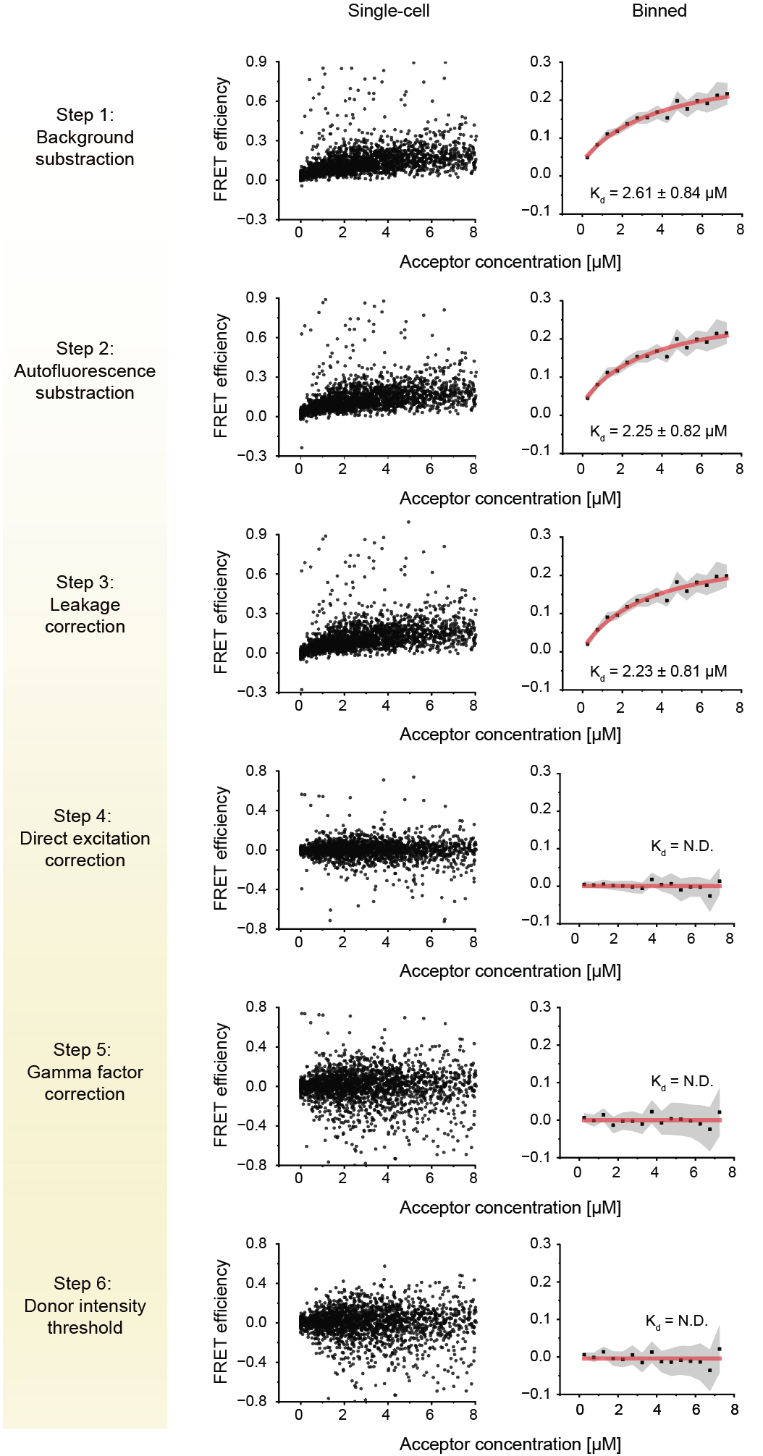


Figure S9. Stepwise correction of FRET efficiency of another negative control.

Single-cell scatter plots (left) and binned scatter plots (right) showing FRET efficiency versus acceptor concentration for the non-interacting protein pair (eGFPd and ^N^DD_MlnC_-mRFP) during a series of correction steps in KD-FRET. Again, the direct excitation correction effectively removes the false-positives. K_d_ values are reported as mean ± standard deviation (SD) of triplicates. The gray-shaded regions represent the 95% confidence intervals for the binned mean FRET efficiency. N.D.: Not Determined.


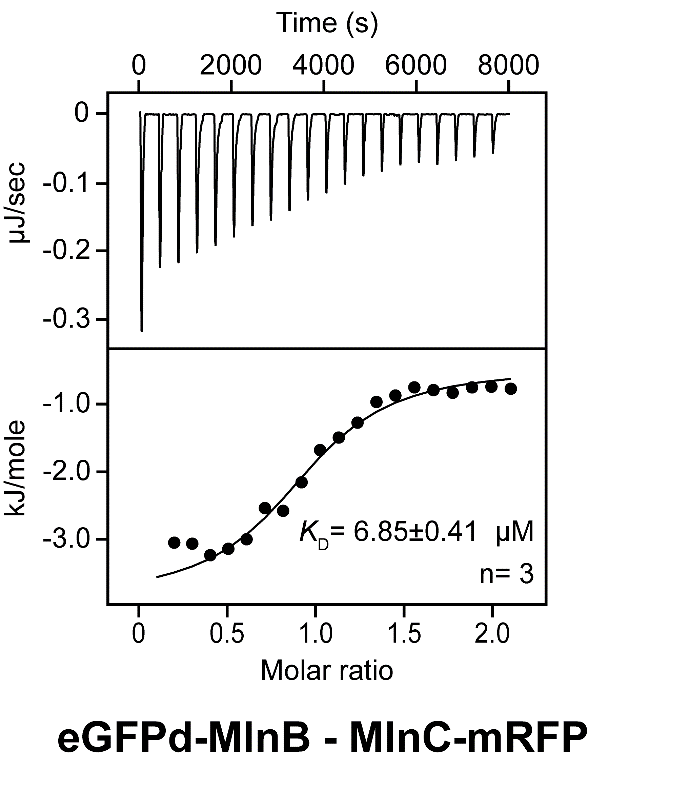


Figure S10. Isothermal titration calorimetry measurements of ^C^DD_MlnB_/^N^DD_MlnC_. Raw thermograms (upper panels) and integrated heats of injection (lower panels) are shown with fitted binding curves. The measured K_d_ values were 6.85 ± 0.41 μM for ^C^DD_MlnB_/^N^DD_MlnC_ pair (mean ± SD). Measurements were performed in triplicate at 37 ºC.


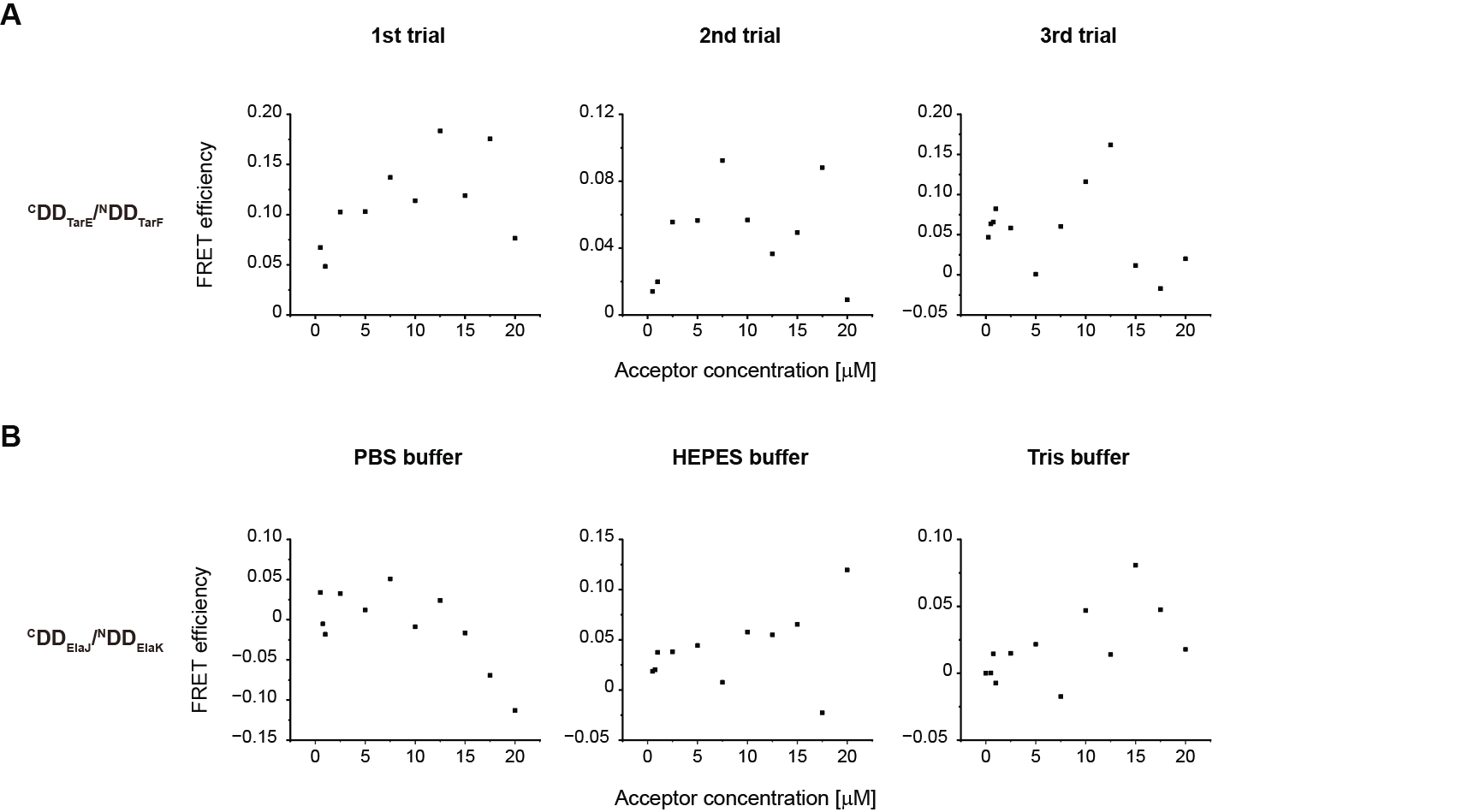


Figure S11. Unstable K_d_ measurements of PPI pairs *in vitro*.

(A) FRET efficiency versus acceptor concentration plots for three independent trials of ^C^DD_TarE_/^N^DD_TarF_, showing inconsistent FRET signals due to the lack of interaction between these pairs *in vitro*. (B) FRET efficiency measurements for ^C^DD_ElaJ_/^N^DD_ElaK_ pairs under different buffer conditions (PBS, HEPES, Tris) also showed inconsistent FRET signals. However, the K_d_ values of ^C^DD_TarE_/^N^DD_TarF_ and ^C^DD_ElaJ_/^N^DD_ElaK_ were successfully measured directly in living cells (Figure 5A). All measurements were performed at 37 ºC.


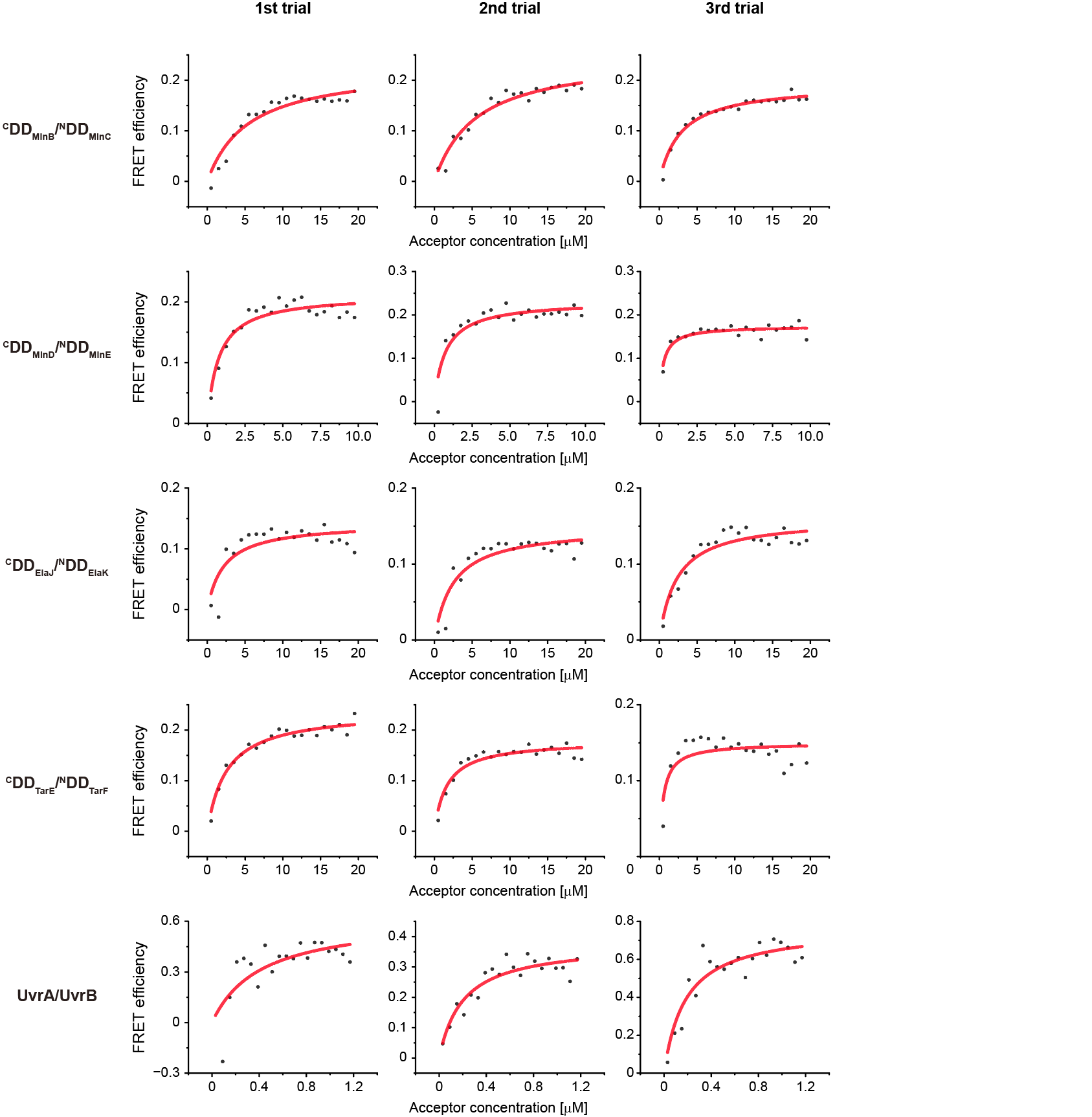


Figure S12. Reproducibility of K_d_ measurements for various PPI pairs using KD-FRET.

FRET efficiency versus acceptor concentration plots for three independent trials of KD-FRET measurements for multiple PPI pairs, including ^C^DD_MlnB_/^N^DD_MlnC_, ^C^DD_MlnD_/^N^DD_MlnE_, ^C^DD_ElaJ_/^N^DD_ElaK_, ^C^DD_TarE_/^N^DD_TarF_, and UvrA/UvrB. The data demonstrate the reproducibility of the KD-FRET method across different trials, with consistent K_d_ values obtained in each experiment. All measurements were performed at 37 ºC.

**Supplementary Tables**

Table S1. Oligonucleotides used for molecular cloning

| **Name** | **Sequence (5' -> 3')** |
| --- | --- |
| **Plasmid cloning** |  |
| pSY011_V_fw | gcatggatgagctgtacaaataaggatccaaac |
| pSY011_V_rv | tcctcgcccttggaaaccatatgtatatctccttcttaaaagatcttttg |
| pSY011_I_fw | tttaagaaggagatatacatatggtttccaagggcg |
| pSY011_I_fw | ccttactcgagtttggatccttatttgtacagctcatccatgc |
| pSY019_V_fw | cgcccttgctcaccat |
| pSY019_V_rv | gcatggacgagctgtacaag |
| pSY019_I_fw | atggtgagcaagggcg |
| pSY019_I_rv | cttgtacagctcgtccatgc |
| pSY026_V_fw | tttaagaaggagatatacatatggtgagcaagggcgag |
| pSY026_V_rv | aggatcttcaccaccggattccagcttgtacagctcgtccatgcc |
| pSY026_I_fw | ctggaatccggtggtgaagatcctatggtttccaagggcgagg |
| pSY026_I_rv | cctcgcccttgctcaccatatgtatatctccttcttaaaagatc |
| pSY028_V_fw | gcatggacgagctgtacaag |
| pSY028_V_rv | tcctcgcccttgctcaccatatgtatatctccttcttaaaag |
| pSY028_I_fw | atggtgagcaagggcg |
| pSY028_I_rv | cttgtacagctcgtccatgc |
| pSY030_V_fw | cgccactccaccggcgcctaaggatccaaactcgagtaaggatc |
| pSY030_V_rv | gacgtcctcggaggaggccataggatcttcaccaccgga |
| pSY030_I_fw | ccggtggtgaagatcctatggcctcctccgagg |
| pSY030_I_rv | actcgagtttggatccttaggcgccggtggag |
| pSY032_V_fw | cgccactccaccggcgcctaaggatccaaactcgagtaaggatc |
| pSY032_V_rv | acgtcctcggaggaggccatatgtatatctccttcttaaaagatcttttg |
| pSY032_I_fw | ctttccggtcgccaccatggcctcctccgagg |
| pSY032_I_rv | actcgagtttggatccttaggcgccggtggag |
| pSY060_fw | taaacgcgtgctagaggcac |
| pSY060_rv | ggcggcggtcacgaa |
| pSY079_fw | taattaattaagggctcgagaagcttgaattc |
| pSY079_rv | ggcggcggtcacgaa |
| pSY087_V_fw | ttcgtgaccgccgccctcgagcaccaccaccac |
| pSY087_V_rv | gcccttgctcaccatatgtatatctccttcttaaagttaaacaaaattatttctagagg |
| pSY087_I_fw | gaaggagatatacatatggtgagcaagggcga |
| pSY087_I_rv | gtggtggtgctcgagggcggcggtcacg |
| pSY106_V_fw | ctccaccggcgcctaagatcc |
| pSY106_V_rv | gcccttgctcaccattcccggggatccac |
| pSY106_I_fw | cgtggatccccgggaatggtgagcaagggcgag |
| pSY106_I_rv | gttagcagccggatcttaggcgccggtggag |
| pSY113_V_fw | agctgctggagggaaaataaacgcgtgctagaggcacg |
| pSY113_V_rv | cctgatcttcatttttgctgccgccgcc |
| pSY113_I_fw | agcggcggcggcagcaaaaatgaagatcaggaaattctcttcttattagaacaattgaaaaacg |
| pSY113_I_rv | tctagcacgcgtttattttccctccagcagctgataaacttgg |
| pSY114_V_fw | agaaaagaacaaatgggcggcggcagc |
| pSY114_V_rv | ttgtttgatctgcatatgtatatctccttcttaaaagatcttttgaattcgg |
| pSY114_I_fw | gaaggagatatacatatgcagatcaaacaaatactttcc |
| pSY114_I_rv | gccgctgccgccgcccatttgttcttttctgtaagtgcgaattagc |
| pSY115_fw | aagaaggagatatacatatggcctcctccgaggacgtc |
| pSY115_rv | gacgtcctcggaggaggccatatgtatatctccttcttaaagttaaacaaaattatttctagaggg |
| pSY120_V_fw | ttgcttttccatgaatgaacgcgtgctagaggcac |
| pSY120_V_rv | tccatctgctgtatcgctgccgccgc |
| pSY120_I_fw | agcggcggcggcagcgatacagcagatggagaaatcatgtcgct |
| pSY120_I_rv | gcctctagcacgcgttcattcatggaaaagcaactcctctatcag |
| pSY121_V_fw | ttggggaaaacccatggcggcggcagc |
| pSY121_V_rv | cgttttttcattcatatgtatatctccttcttaaaagatcttttgaattcgg |
| pSY121_I_fw | gagatatacatatgaatgaaaaaacgatgcaaattgagc |
| pSY121_I_rv | gccgctgccgccgccatgggttttccccaagctct |
| pSY140_V_fw | gtgaccgccgcctaaacgcgtgctagaggcac |
| pSY140_V_rv | ttcgatcttatccatggatcccctcctggg |
| pSY140_I_fw | cccaggaggggatccatggataagatcgaagttcggggcgccc |
| pSY140_I_rv | gcctctagcacgcgtttaggcggcggtcacg |
| pSY141_V_fw | cactccaccggcgcctaa |
| pSY141_V_rv | gaacggtttactcatatgtatatctccttcttaaaagatcttttgaattcggt |
| pSY141_I_fw | ggagatatacatatgagtaaaccgttcaaactgaattccgc |
| pSY141_I_rv | ctcgagtttggatccttaggcgccggtggag |
| pSY142_V_fw | gcggcagcaaagaaaacgatccgctg |
| pSY142_V_rv | ttttctttgctgccgccgccgctgcc |
| pSY142_I_fw | ttcacctcaggaggggatcc |
| pSY142_I_rv | ttgatgcctctagcacgcgtgcctctagcacgcgtttaattggcatttcttat |
| pSY143_V_fw | acgtcgtgggcggcggcagcggcggc |
| pSY143_V_rv | ccgccgcccacgacgttgcccttact |
| pSY143_I_fw | agaaggagatatacatatgaaagattatatcgaacatatactcgaagagataaaacacaaccgtcttagtaaacaggag |
| pSY143_I_rv | ccttactcgagtttggatccttaggcgccggtggagtg |
| pSY148_V_fw | gcggcagcgacgaagaacagcattgg |
| pSY148_V_rv | tcttcgtcgctgccgccgccgctgcc |
| pSY148_I_fw | ttcacctcaggaggggatcc |
| pSY148_I_rv | ttgatgcctctagcacgcgtgcctctagcacgcgttcataactgaatcgcctc |
| pSY149_V_fw | agcagcagggcggcggcagcggcggc |
| pSY149_V_rv | ccgccgccctgctgctgaagttgttt |
| pSY149_I_fw | agaaggagatatacatatgggaatgatcgataaagacgccattctggcggcggtaaagtcgggtgagttaacag |
| pSY149_I_rv | ccttactcgagtttggatccttaggcgccggtggagtg |
| pSY159_fw | ctggaatccggtggtgaagatc |
| pSY159_rv | ggcggcggtcacgaa |
| pSY165_fw | gctcagtcctaggtataatgctagctaaagagacggaatccagtggttttagagctagaaatagcaagt |
| pSY165_rv | tagctgtcaatactctttttgaggcg |
| pSY166_fw | gctcagtcctaggtataatgctagcggcgcgtaaagagacactgggttttagagctagaaatagc |
| pSY166_rv | tagctgtcaatactctttttgaggcg |
| pSY171_V_fw | cggcgcctaattaattaagggctcga |
| pSY171_V_rv | tgtgatcgcgcttctcgt |
| pSY171_I_fw | acgagaagcgcgatcaca |
| pSY171_I_rv | cttctcgagcccttaattaattaggcgccggtggag |
|  |  |
| ***In vitro* FRET** |  |
| pSY172,4_I1_fw | tgccgcgcggcagccatatggtgagcaagggcgagga |
| pSY172,4_I1_rv | ctccaccgccggcggcggtcacgaactcca |
| pSY172,4_I2_fw | tgaccgccgccggcggtggagggtcggga |
| pSY172_I2_rv | agcagccggatcctcgagttattttccctccagcag |
| pSY174_I2_rv | gttagcagccggatcctcattcatggaaaagcaa |
| pSY173_I1_fw | taagaaggagatatacatatgcagatcaaacaaata |
| pSY175_I1_fw | taagaaggagatatacatatgaatgaaaaaacgatg |
| pSY173,5_I1_rv | tcggaggaggcggaacctccgcctcccgat |
| pSY173,5_I2_fw | aggttccgcctcctccgaggacgtcat |
| pSY173,5_I2_rv | tggtggtggtggtgctcgagggcgccggtggagtggcg |
|  |  |
| **In-cell flavonoid assay** |  |
| 6k-4CL_fw | agaaggagatatacatatggcgccacaagaacaa |
| 4CL-rbs_rv | tttggcggccgcttacaatccatttgctag |
| rbs-CHS_fw | taagcggccgccaaaagatcttttaagaaggagatatatctagatggtgacagtcgaggag |
| CHS-6k_rv | ccttactcgagtttggatcctcaagtagcaacactgtg |
| 4CL-MlnB-rbs_rv | tttggcggccgcttattttccctccagcag |
| rbs-MlnC-CHS_fw | taagcggccgccaaaagatcttttaagaaggagatatatctagatgcagatcaaacaaata |
| 4CL-MlnD-rbs_rv | tttggcggccgcttattcatggaaaagcaactc |
| rbs-MlnE-CHS_fw | taagcggccgccaaaagatcttttaagaaggagatatatctagatgaatgaaaaaacgatg |
|  |  |
| **In-cell lycopene assay** |  |
| MlnB-ATG F | gcgctctagaatgaaaaatgaagatcaggaaattctcttcttattag |
| MlnB_G3S*2_R | catggaacctccgcccgatccacctccttttccctccagcagctgataaac |
| crtE_G3S*2_F | ggaggtggatcgggcggaggttccatggattacgcgaacatcctcaca |
| crtE_R | atcgtcgactcacagagggatatcggctagc |
| MlnC_nDD_F | gcgactagtatgcagatcaaacaaatactttccttaattgaag |
| (G4S)*2 R | ggaacctccgcctcccgatccacctccacc |
| erg20_G4S*2_F | tggaggtggatcgggaggcggaggttccatggcttcagaaaaagaaattaggagagag |
| erg20_R | gcgctcgagttatttacttctcttgtaaaccttgttcaaaaacg |
|  |  |
| **Lambda red recombination** |  |
| pBAD_Tsr_F | ggaattgtgagcggataacaatttcacacaggaaacagctatgttaaaacgtatcaaaattgtgaccagc |
| pBAD_Tsr_R | taggcctgataagcgcagcgtatcaggcaatttttataatacagtaagtgtaggctggagc |
|  |  |
| **Cas9 editing template** |  |
| templateDNA_UvrA | aatactgtatattcattcaggtcaatttgtgtcataattacgaagcatcacacacggcacgcttccttaagccgatgctg |
| templateDNA_UvrB | aatttgttggcataattaagtacgacgagtaaaattacatccagttgcatcagctgcgtgagctgtttatcgcggcatcg |

Table S2. Plasmids used in this study

| **Plasmid** | **Backbone** | **Description** | **Source** | **Note** |
| --- | --- | --- | --- | --- |
| pETcoco-1 | pETcoco-1 |  | Novagen #71129 | Single vector system for in-cell FRET measurement |
| pSY011 | pBBR6k | pBBR6k-mCherry | This study |  |
| pSY019 | pFF838 | pFF838-eGFP | This study |  |
| pSY026 | pBBR6k | pBBR6k-eYFP-mCherry | This study |  |
| pSY028 | pBBR6k | pBBR6k-eGFP-mCherry | This study |  |
| pSY030 | pBBR6k | pBBR6k-eGFP-mRFP | This study |  |
| pSY032 | pBBR6k | pBBR6k-mRFP | This study | Negative control for in-cell FRET measurement |
| pSY060 | pFF838 | pFF838-eGFPd | This study | Negative control for in-cell FRET measurement |
| pSY079 | pMRBAD | pMRBAD-Tsr-eGFPd | This study | Tsr-eGFPd recombination |
| pSY087 | pET21c | pET21c-eGFPd-6His | This study | Negative control for *in vitro* FRET measurement |
| pSY106 | pET21c | pET21c-GST-eGFPd-mRFP-6His | This study | Positive control for *in vitro* FRET measurement |
| pSY113 | pFF838 | pFF838-eGFPd-(GGGS)_2_-MlnB-cDD | This study |  |
| pSY114 | pBBR6k | pBBR6k-MlnC-nDD-(GGGS)_2_-mRFP | This study |  |
| pSY115 | pET21c | pET21c-mRFP-6His | This study | Negative control for *in vitro* FRET measurement |
| pSY120 | pFF838 | pFF838-eGFPd-(GGGS)_2_-MlnD-cDD | This study |  |
| pSY121 | pBBR6k | pBBR6k-MlnE-nDD-(GGGS)_2_-mRFP | This study |  |
| pSY140 | pFF838 | pFF838-UvrA-(GGGGS)_2_-eGFPd | This study |  |
| pSY141 | pBBR6k | pBBR6k-UvrB-(GGGS)_2_-mRFP | This study |  |
| pSY142 | pFF838 | pFF838-eGFPd-(GGGS)_2_-ElaJ-cDD | This study |  |
| pSY143 | pBBR6k | pBBR6k-ElaK-nDD-(GGGS)_2_-mRFP | This study |  |
| pSY148 | pFF838 | pFF838-eGFPd-(GGGS)_2_-TarE-cDD | This study |  |
| pSY149 | pBBR6k | pBBR6k-TarF-nDD-(GGGS)_2_-mRFP | This study |  |
| pSY159 | pBBR6k | pBBR6k-eGFPd-mRFP | This study | Positive control for in-cell FRET measurement |
| pSY165 | pCDF | pCDF-UPJ23119-uvrA-sgRNA | This study | Cas9 editing for UvrA deletion BW mutant |
| pSY166 | pCDF | pCDF-UPJ23119-uvrB-sgRNA | This study | Cas9 editing for UvrB deletion BW mutant |
| pSY171 | pMRBAD | pMRBAD-Tsr-eGFPd-mRFP | This study | Tsr-eGFPd-mRFP recombination |
| pMRBAD-Tsr-mRFP | pMRBAD | pMRBAD-Tsr-mRFP | This study | Tsr-mRFP recombination |
| pKD46 | pKD46 |  | Datsenko *et al*.^[5]^ | Lambda red recombination |
| pCas-CDF | pCas | pCas-CDF (pCas-Ptrc-sgRNA-pMB1::sgRNA-pCDF) | Seok *et al*. ^[6]^ | Cas9 editing |
| pCDF-sg | pCDF | pCDF-P_BBa_K2753055_-sgRNA | Seok *et al*. ^[6]^ | Cas9 editing |
| pcDNA3.1(+) eGFP | pcDNA3.1 | pcDNA3.1-eGFP | Xiao *et al*. ^[7]^ |  |
| pcDNA3-mRFP | pcDNA3 | pcDNA3-mRFP | Addgene #13032 |  |
| pFF838 | pFF838 | pFF838 | Farzadfard *et al*.^[8]^ | Donor expression vector for in-cell FRET measurement |
| pBBR6k-GFPuv | pBBR6k | pBBR6k-GFPuv | Cook *et al*.^[9]^ | Acceptor expression vector for in-cell FRET measurement |
|  |  |  |  |  |
| pSY172 | pET15b | pET15b-6His-eGFPd-(GGGS)_2_-MlnB-cDD | This study | *In vitro* FRET measurement |
| pSY173 | pET21c | pET21c-MlnC-nDD-(GGGS)_2_-mRFP-6His | This study | *In vitro* FRET measurement |
| pSY174 | pET15b | pET15b-6His-eGFPd-(GGGS)_2_-MlnD-cDD | This study | *In vitro* FRET measurement |
| pSY175 | pET21c | pET21c-MlnE-nDD-(GGGS)_2_-mRFP-6His | This study | *In vitro* FRET measurement |
| pNAR_Free_ | pBBR6k | pBBR6k-4CL / CHS | This study | Control for in-cell flavonoid assay |
| pNAR_MlnB_ | pBBR6k | pBBR6k-4CL-(GGGGS)_2_-MlnB-cDD / CHS | This study | Donor control for in-cell flavonoid assay |
| pNAR_MlnC_ | pBBR6k | pBBR6k-4CL / MlnC-nDD-(GGGGS)_3_-CHS | This study | Acceptor control for in-cell flavonoid assay |
| pNAR_MlnB-C_ | pBBR6k | pBBR6k-4CL-(GGGGS)_2_-MlnB-cDD / MlnC-nDD-(GGGGS)_3_-CHS | This study | In-cell flavonoid assay |
| pNAR_MlnD_ | pBBR6k | pBBR6k-4CL-(GGGGS)_2_-MlnD-cDD / CHS | This study | Donor control for in-cell flavonoid assay |
| pNAR_MlnE_ | pBBR6k | pBBR6k-4CL / MlnE-nDD-(GGGGS)_3_-CHS | This study | Acceptor control for in-cell flavonoid assay |
| pNAR_MlnD-E_ | pBBR6k | pBBR6k-4CL-(GGGGS)_2_-MlnD-cDD / MlnE-nDD-(GGGGS)_3_-CHS | This study | In-cell flavonoid assay |
| pLYC_Free_ | p416GPD | CrtB / CrtE / ERG20 | This study | Control for in-cell lycopene assay |
| pLYC_MlnB_ | p416GPD | CrtB / MlnB-cDD-(GGGS)_2_-CrtE / ERG20 | This study | Donor control for in-cell lycopene assay |
| pLYC_MlnC_ | p416GPD | CrtB / CrtE / MlnC-nDD-(GGGGS)_2_-ERG20 | This study | Acceptor control for in-cell lycopene assay |
| pLYC_MlnB-C_ | p416GPD | CrtB / MlnB-cDD-(GGGS)_2_-CrtE / MlnC-nDD-(GGGGS)_2_-ERG20 | This study | In-cell lycopene assay |

Table S3. Strains used in this study

| **Strain** | **Genotype** | **Source** |
| --- | --- | --- |
| BW25993 | F^-^ Δ(rhaD-rhaB)567 ΔlacZ4787(::rrnB-3) λ- rph-1 Δ(rhaD-rhaB)568 hsdR514 | Datsenko *et al*.^[5]^ |
| SY002 | BW ΔlacZ::tsr-egfpd | This study |
| SY004 | BW ΔlacZ::tsr-egfpd-mRFP | This study |
| SY006 | BW ΔuvrA ΔuvrB | This study |
| SY007 | BW ΔlacZ::tsr-mrfp | This study |
| BL21(DE3) | F- ompT hsdSB (rB–, mB–) gal dcm (DE3) | Invitrogen #C600003 |
| NAR_Free_ | BW25993 harboring pNAR_Free_ | This study |
| NAR_MlnB_ | BW25993 harboring pNAR_MlnB_ | This study |
| NAR_MlnC_ | BW25993 harboring pNAR_MlnC_ | This study |
| NAR_MlnB-C_ | BW25993 harboring pNAR_MlnB-C_ | This study |
| NAR_MlnD_ | BW25993 harboring pNAR_MlnD_ | This study |
| NAR_MlnE_ | BW25993 harboring pNAR_MlnE_ | This study |
| NAR_MlnD-E_ | BW25993 harboring pNAR_MlnD-E_ | This study |
| CEN.PK2-1C | MATa his3D1 leu2-3_112 ura3-52 trp1-289 MAL2-8c SUC2 | EUROSCARF |
| LYC | CEN.PK2-1C *H4::*P*_TEF_*-*tHMG1*-T*_ADH1_*  H8::P*_GPD_*-*crtI*-T*_CYC1_* | This study |
| LYC_Free_ | LYC harboring pLYC_Free_ | This study |
| LYC_MlnB_ | LYC harboring pLYC_MlnB_ | This study |
| LYC_MlnC_ | LYC harboring pLYC_MlnC_ | This study |
| LYC_MlnB-C_ | LYC harboring pLYC_MlnB-C_ | This study |

**Supplementary References**

[1] J. Yu, J. Xiao, X. Ren, K. Lao, X. S. Xie, *Science* **2006**, *311*, 1600-1603.

[2] S. Yang, S. Kim, D.-K. Kim, H. Jeon An, J. Bae Son, A. Hedén Gynnå, N. Ki Lee, *Nat. Commun.* **2019**, *10*, 5131.

[3] H. Shi, Y. Hu, P. D. Odermatt, C. G. Gonzalez, L. Zhang, J. E. Elias, F. Chang, K. C. Huang, *Nat. Commun.* **2021**, *12*, 1975.

[4] A. Mitraki, B. Fane, C. Haase-Pettingell, J. Sturtevant, J. King, *Science* **1991**, *253*, 54-58.

[5] K. A. Datsenko, B. L. Wanner, *Proc. Natl. Acad. Sci. U.S.A.* **2000**, *97*, 6640-6645.

[6] J. Y. Seok, Y. H. Han, J. S. Yang, J. Yang, H. G. Lim, S. G. Kim, S. W. Seo, G. Y. Jung, *Cell Rep.* **2021**, *36*, 109589.

[7] M. S. Xiao, J. E. Wilusz, *Nucleic Acids Res.* **2019**, *47*, 8755-8769.

[8] F. Farzadfard, T. K. Lu, *Science* **2014**, *346*, 1256272.

[9] T. B. Cook, J. M. Rand, W. Nurani, D. K. Courtney, S. A. Liu, B. F. Pfleger, *J. Ind. Microbiol. Biotechnol.* **2018**, *45*, 517-527.
